# Understanding Molecular Excited States at the Metal–Molecule Interface via Transition Density Matrix Analysis—A Case Study of Azobenzene Thiols on Gold

**DOI:** 10.1021/acsphyschemau.6c00046

**Published:** 2026-06-12

**Authors:** Nicolas Jahn, Evgenii Titov

**Affiliations:** Institute of Chemistry, 26583 University of Potsdam, Karl-Liebknecht-Str. 24-25, 14476 Potsdam-Golm, Germany

**Keywords:** azobenzene, gold, excited state, DFT, DFTB, transition density matrix

## Abstract

Noble metal nanoparticles are rapidly gaining popularity as novel catalytic platforms to influence chemical reactions in various ways. Despite an increasingly large number of studies, many key processes especially at the metal–molecule interface are fundamentally still not fully understood up to this day. Throughout this work, we present a systematic study targeting the molecular excited states of covalently linked azobenzenes (ABs) on gold via a combination of time-dependent density functional theory and tight-binding calculations with transition density matrix analysis. We find that the optically bright ππ* state is strongly resonant to close-lying local gold excitations, which leads to splitting and redshift of the ππ* state and increased UV/vis absorption around the ππ* excitation energy. We show that these findings hold true across different AB isomers and demonstrate how a systematic variation of the AB–gold distance leads to a gradual localization of the ππ* excitation. Moreover, we discuss how a direct electronic interaction between the AB and the gold surface leads to the formation of delocalized hybrid states and investigate the exciton formation of AB dimers at the metal interface. Our results are carefully verified across a wide range of computational parameters including a large number of different density functionals, basis sets, and gold clusters of varying forms and sizes. The presented workflow is easily applicable to other functional molecules on metal surfaces to further broaden the understanding of substrate–surface interactions at the interface.

## Graphical abstract

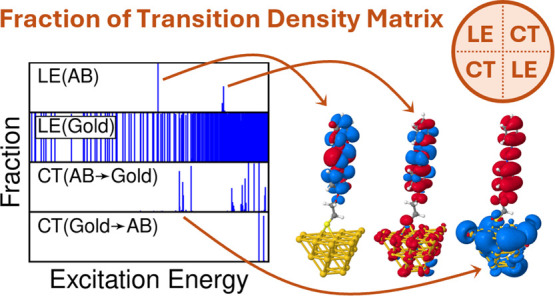

## 1 Introduction

For decades, numerous types of catalysts have been employed both in scientific research and industrial applications to influence chemical reactions in various ways. Catalysis allows one to obtain wanted reaction products and/or to suppress unwanted side reactions/products, to accelerate chemical transformations, to increase reaction yields, and to operate at milder reaction conditions.^1–3^ In recent years, noble metal nanoparticles (NPs) have gained significant attention as novel catalytic systems, utilizing their localized surface plasmon resonances (LSPR) and opening the field of plasmon-driven chemistry. Thereby, locally enhanced electric fields, increased temperatures, and hot charge carriers, as observed after LSPR excitation, may all influence chemical reactions. Since LSPR bands are typically found in the visible to near-UV range, they potentially allow for the use of environmentally friendly light sources, ideally sunlight.^4–7^

Although many attempts have already been made in the literature to employ plasmonic effects in catalysis, the number of successful cases is still surprisingly low owing to the immense complexity of the studied systems.^8–10^ Especially the processes directly occurring at the metal–molecule interface, the center at which chemical transformations take place, remain challenging to grasp on a microscopic level up to this day. As a result, even the mechanisms of established model reactions such as the vastly studied dimerization of *para-*nitrothiophenol remain highly debated in the literature.^11–15^

Molecular switches, e.g., azobenzenes (ABs) and related derivatives, are another intriguing class of molecules, which have been extensively studied on metal NPs. In particular, various groups have reported a presumably hole-transfer catalyzed thermal *cis* → *trans* isomerization process of ABs in the vicinity of gold NPs in past studies.^16–18^ On the other hand, the photoisomerization behavior of ABs on plasmonic NPs is arguably more complicated. In many experimental studies, the photoswitching of metal-bound ABs remained mostly unchanged in comparison to the free AB in solution apart from possible quenching at short AB–metal distances.^19–25^ However, it has also been shown that a high density of ABs on the metal surface can lead to steric hindrance of the photoswitching process.^26^ Moreover, tailor-made spacers can even accelerate the isomerization of ABs on Au(111), possibly via a new metal-mediated intersystem crossing pathway.^27^

One of the key molecular properties to better understand the chemical behavior of different reactants at plasmonic NPs is their electronically excited states, relevant, e.g., for charge and energy transfer processes after LSPR excitation.^28^ In this regard, we consider ABs as valuable model systems due to their vastly different and well-known low-lying excited states, namely, consisting of an optically dark nπ* (S_1_) and an optically bright ππ* (S_2_) state.^29^ To broaden the understanding of substrate–surface interactions at the metal–molecule interface, we thus aim to systematically investigate the excited states of covalently linked AB thiols on gold from a computational standpoint in the present work. Specifically, we want to understand how the AB excited states behave at varying distances to the metal surface, how the relative orientation of the molecule to the metal and the AB isomer (*trans* or *cis*) influences the excited states, as well as how metal-bound AB dimers form molecular excitons. Although ABs have been theoretically investigated on metal surfaces in the past,^30–35^ no systematic study comparable to this present work is, to the best of our knowledge, currently available in the literature.

Since excited states of bare metal or hybrid metal–molecule nanostructures are computationally demanding within a quantum chemical framework, time-dependent density functional theory (TD-DFT) and density functional tight-binding (TD-DFTB) are currently considered as the main workhorse methods in computational plasmonics.^36–41^ The popularity of TD-DFT in the field has further led to efficient new developments in recent years including the TD-DFT+TB method by Asadi-Aghbolaghi et al.^42^ and the complex polarizability TD-DFT method by Baseggio et al.^43^ In particular, the latter approach has recently been found to produce results in excellent agreement with experimental findings.^44^ Apart from the choice of a suitable computational method, an efficient way to analyze large numbers of excited states is often required as even small metal nanostructures already have hundreds of excited states in the visible to near-UV range. We aim to meet this challenge by combining time-dependent linear-response DFT and DFTB calculations with transition density matrix (TDM) analysis^45–49^ to characterize the dense excited-state manifolds of our metal-bound AB systems. Although TDM analysis has been extensively used in the past, e.g., for describing charge-transfer (CT) excitations and exciton formation,^50–53^ its efficiency has rarely been highlighted for hybrid metal–molecule systems thus far. Moreover, TDM analysis provides a basis to directly compare computational parameters such as the influence of the chosen density functional and basis set or the form and size of the approximate metal cluster model on the computed excited states. As the second goal, we aim to also discuss these points throughout this work.

## 2 Computational Details

### 2.1 Choice of Structural Models

Owing to the structural diversity of hybrid AB–gold models considered in this work, we begin by introducing our chosen models in this section. An overview of the considered structures is shown in Figure 1, which is divided into the investigated molecular parameters mentioned in the introduction. Further models are shown and discussed in the results section of this work and in the Supporting Information (SI).

**Figure 1:**
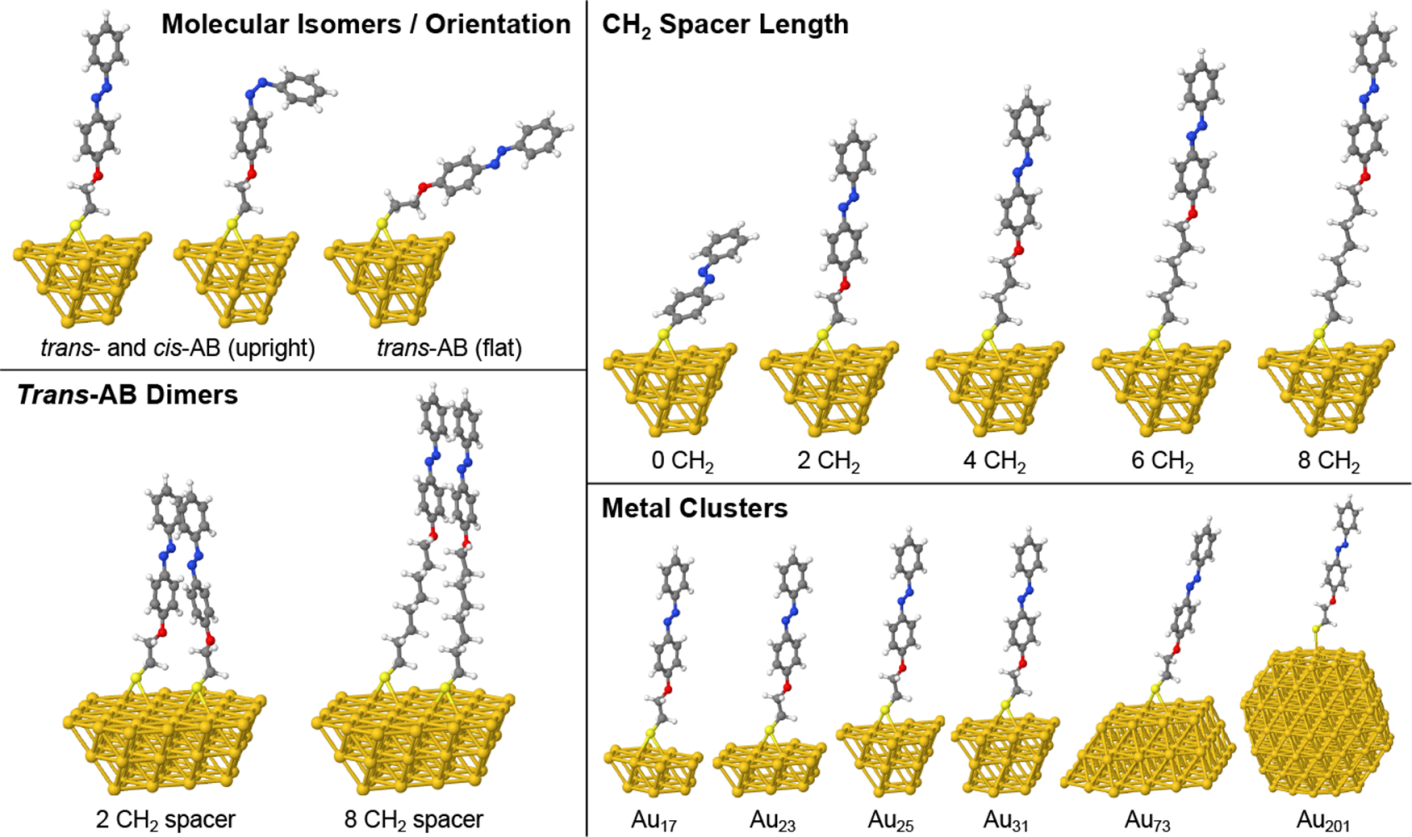
Overview of the different covalently linked hybrid AB–gold models employed in this work. Top left: upright *trans*-AB/*cis*-AB and flat *trans*-AB isomers on Au_25_. Top right: upright *trans*-AB with a varying number of CH_2_ spacer units on Au_25_. Bottom left: upright *trans*-AB dimers with a short (two CH_2_ units) and long (eight CH_2_ units) alkyl spacer on Au_64_. Bottom right: upright *trans*-AB with two CH_2_ units on different gold clusters. Geometry color code: Gray: C, white: H, blue: N, red: O, yellow: S, and gold: Au.

Following related computational studies in the literature,^39–41^ we approximately represent the metal part of our hybrid systems by various cluster models as depicted in Figure 1. The specific gold clusters were chosen based on a set of general criteria: (i) the cluster surface follows the Au(111) structure and all clusters should consist of at least three layers to represent the ABC layered Au(111) pattern, although some two-layered test systems have also been used. (ii) The ABs are covalently linked to the gold surface via a deprotonated thiolate group with the sulfur atom binding to “surface-like” gold atoms, namely, gold atoms with nine nearest neighbors. (iii) The cluster structures remain frozen with a nearest neighbor Au–Au distance of 2.876 Å except for test cases with an unfixed first gold layer. The Au–Au distance was obtained from a periodic bulk optimization of gold (see SI, Section S1) using the TPSS^54^ functional in VASP 5.4.4.^55–57^ (iv) The hybrid models are well represented by closed-shell singlet ground states without self-consistent field (SCF) instabilities toward possible unrestricted solutions. The validity of (iv) has been confirmed via TPSS calculations using higher-spin multiplicity and RHF → UHF wave function stability analysis. We note that structural rearrangements of the gold surface due to the binding ABs are generally neglected based on point (iii) to preserve the atomic structure of the clusters during geometry relaxations. We plan to address this point in greater detail in a second, related study in the near future focusing on the excited-state nuclear dynamics of hybrid AB–gold systems. A majority of calculations in this work employ the three-layered Au_25_ cluster model highlighted in Figure 1, which fulfills all of the mentioned criteria at a moderate computational cost. Other cluster models have primarily been used to investigate the consistency of the computational results with regard to the form and size of the metal cluster and for the AB dimer models (Figure 1 bottom left), which require a larger surface area.

From a molecular side, we consider both the energetically favored *trans*-AB as well as the *cis*-AB isomers in two different orientations relative to the cluster surface: (i) A perpendicular (“upright”) orientation, which resembles a SAM-like (self-assembled monolayer) arrangement of the ABs as experimentally expected at high surface coverages^58–60^ and (ii) a horizontal (“flat”) orientation, which acts as an approximate model for low surface coverage cases. The AB–gold distance is varied by introducing alkyl spacers consisting of *N* individual CH_2_ groups (*N* = 2, 4, 6, or 8), which are bound to the metal surface via a deprotonated thiolate group and connected to the AB chromophores via an ether oxygen atom. The structural pattern of the AB, the linker, and the spacer was chosen to match the synthesized AB samples in related experimental studies.^19–25^ Moreover, a *trans*-AB thiol directly bound to the metal surface via the sulfur atom has also been considered. The AB dimer models shown in Figure 1 have been aligned in parallel due to π–π stacking interactions of neighboring *trans*-ABs with the same spacer length.

### 2.2 Geometry Relaxations and Excited-State Calculations

All DFT calculations in this work have been performed within a restricted formalism in vacuum employing ORCA 6.^61–63^ The computed structures were built from scratch and subsequently optimized using the meta-generalized gradient approximation (*meta*-GGA) functional TPSS^54^ within the resolution-of-identity (RI) approximation paired with def2-TZVP^64^ basis and def2/J^65^ auxiliary basis sets. For geometry optimizations of hybrid AB–gold models, the metal clusters were kept frozen during the relaxation, and only the molecular parts including linker (S atom) and spacer groups were fully optimized. To investigate the geometrical changes of the cluster surfaces due to the bound ABs, calculations with an unfixed first metal layer have also been performed in some cases. The AB dimer models on gold were constructed in three steps by first relaxing two S–CH_3_ groups on the Au_64_ cluster (see Figure 1 bottom left) to obtain the binding positions of the ABs. The metal cluster was subsequently removed, the S–CH_3_ groups were kept fixed, and the sulfur atoms saturated with a hydrogen atom and the remaining CH_2_ spacers together with the *trans*-ABs were added and optimized at the TPSS level including Grimme’s D3 dispersion correction^66^ with Becke–Johnson damping (D3BJ).^67^ Afterward, the optimized dimer structures were again linked to the metal cluster and used for further calculations. Most of the computations were carried out using very tight SCF and geometry convergence settings paired with the DEFGRID3 numerical integration grid as implemented in ORCA. For computational reasons, calculations involving the larger Au_64_ and Au_73_ clusters were performed with the smaller def2-SVP basis set, tight convergence settings, and DEFGRID2 instead. To approximately account for relativistic effects of gold, def2 effective core potentials (ECPs)^68^ were used to replace the 60 inner core electrons of each gold atom.

Local minima on the potential energy surfaces were confirmed by vibrational frequency analysis. In the case of AB–gold hybrid models, only the molecular modes of the optimized AB including the linker and the spacer were analyzed. SCF stability analysis was performed on all optimized structures to check for potential instabilities toward unrestricted solutions. Furthermore, single point energies with triplet spin multiplicity were also computed to check the viability of the restricted DFT formalism as some cluster geometries tend to energetically prefer higher-spin multiplicities.

Electronically excited states were computed using both linear-response TD-DFT^69^ as well as the Tamm–Dancoff approximation (TDA)^70^ in ORCA on the TPSS-optimized geometries. For AB on the Au_25_ cluster, 250 singlet excited states each (300 each for *cis*-ABs) were computed using three different range-separated hybrid functionals, namely, CAM-B3LYP,^71^ ωB97X,^72^ and ωB97X-D3.^73^ We further employed configuration interaction singles (CIS) as a wave function based alternative to the TDA. For the *trans*-ABs on Au_25_, 350 excited states each were also computed using the global hybrid functional PBE0 ^74^ as well as 600 states each using the GGA functional PBE.^75^ The numbers of excited states were chosen to always include both the low-lying nπ* and ππ* states of both AB isomers in the computed energy ranges. The excited-state calculations were performed using the def2-TZVP basis and def2/J auxiliary basis sets. All hybrid functionals were further used within the RI approximation for the Coulomb part, while seminumerical integration via the chain of spheres algorithm^76^ (RIJCOSX in ORCA) was applied for the exact (Hartree–Fock) exchange.

For *trans*-AB with two CH_2_ alkyl spacers on the other, small gold clusters (Au_17_–Au_31_), 200–350 excited states each were calculated exclusively using CAM-B3LYP employing both TD-DFT and TDA-DFT. Because of computational limitations, calculations using the larger Au_64_ and Au_73_ cluster models were only performed within the TDA and with the smaller def2-SVP basis set. For the AB dimers on Au_64_, 1000 excited states each were computed, whereas 1200 excited states were calculated for *trans*-AB on Au_73_.

Further computational parameters were exclusively tested for *trans*-AB with two CH_2_ spacer groups on Au_25_ using the CAM-B3LYP functional within the TDA. The basis set dependence of the computed excited states was tested within the def2 basis set family employing def2-SVP, def2-TZVPP, and def2-QZVP basis sets, the latter paired with a decontracted def2/J auxiliary basis set. Auxiliary basis set errors of def2/J were tested by pairing the def2-TZVP basis set with a large, on-the-fly generated auxiliary basis obtained via ORCA's automatic auxiliary basis set generation procedure AutoAux.^77^ Further basis set tests were performed using dhf-TZVP paired with dhf ECPs^78^ for gold as well as LANL2DZ and LANL2TZ­(f) basis sets with HayWadt ECPs^79–81^ for gold and a 6-31G* basis set^82,83^ for the organic part. All non-def2 basis sets were also used together with AutoAux generated auxiliary basis sets. Moreover, we tested an all-electron zero-order regular approximation (ZORA) approach^84^ together with ZORA-def2-TZVP basis (SARC-ZORA-TZVP for gold) and SARC/J auxiliary basis sets^85^ to investigate the applicability of the utilized ECPs.

As a lower cost alternative to regular DFT, we also investigated the applicability of semiempirical TD-DFTB as implemented in DFTB+ 24.1 ^86^ employing the auorg-1-1 parameter set.^87–89^ For AB on Au_25_, 800 excited states each (1000 for *cis*-ABs) were computed on the TPSS-optimized geometries, while 5000 excited states each were computed for the *trans*-AB dimers on Au_64_. Furthermore, calculations involving the large Au_201_ cluster model (see Figure 1 bottom right) were exclusively performed at the TD-DFTB level. All corresponding models were fully relaxed including the metal cluster, and 20,000 excited states each were subsequently computed. All TD-DFTB computations were carried out within a truncated excitation space, in which only single-particle excitations with energy differences below the approximate excitation energy of the final requested excited state plus 2 eV (plus 1 eV for Au_201_) were included.

The obtained UV/vis stick spectra in this study were broadened by Gaussians with a broadening parameter of 0.1 eV to obtain vertical electronic spectra. Excited-state characters were mainly determined via a fraction of transition density matrix (FTDM) approach,^49^ which is described in greater detail in the next section. Natural transition orbital (NTO)^90^ and charge density difference (CDD) analyses have also been performed in some cases, for which built-in tools from ORCA and DFTB+ together with Multiwfn 3.8 ^91,92^ were used. NTOs and CDDs have been visualized in Jmol.^93^

### 2.3 TDM Analysis of the Excited States

Excited states of molecular systems are routinely described using orbital-based descriptors, e.g., by directly analyzing relevant molecular orbitals, NTOs, or similar alternatives. However, such “visual” analyses can quickly become impractical when dealing with dense excited-state manifolds and/or many relevant orbital pairs. Different approaches such as transition contribution maps^94^ or individual component maps^95,96^ have thus been employed in past studies to characterize the excited states of complex systems with many orbitals. We address the issue in this work by directly analyzing the ground- to excited-state TDM to numerically quantify the location of an individual excitation on specific atoms or fragments of the system in question.^45,47,48^ The present workflow closely follows the mathematical background of ref 49 and further references therein and is efficiently applicable to large systems and many excited states as investigated in this work.

In a CIS (or TDA-DFT) framework, the TDM within an atomic orbital basis **P** ^[AO]^ can be calculated from the CI expansion coefficients *C*_ov_ of the transitions from occupied (o) to virtual (v) molecular orbitals and the molecular orbital coefficients *c*_μω_ (where μ and ω are atomic orbital indices). Assuming that the sum of squares of *C*_ov_ is normalized to 1/2, the element *P*_μω_^[AO]^ of **P** ^[AO]^ is given by eq 1.^49^

$$P_{\mu \omega }^{\left[\mathrm{AO}\right]}=2\sum_{o}\sum_{v}C_{\mathrm{ov}}c_{\mu o}c_{\omega v}$$ 1

In case of TD-DFT and TD-DFTB, both excitation *C*_o→v_ and de-excitation *C*_o←v_ coefficients need to be taken into account, and the corresponding element *P*_μω_^[AO]^ becomes eq 2.

$$P_{\mu \omega }^{\left[\mathrm{AO}\right]}=2\sum_{o}\sum_{v}C_{o\to v}c_{\mu o}c_{\omega v}+2\sum_{o}\sum_{v}C_{o\leftarrow v}c_{\mu o}c_{\omega v}$$ 2

Multiple variations now exist to further contract **P** ^[AO]^ to fragments of the system of interest, which gives insight into local excitations (LEs) within and CT contributions between the individual fragments. We only employ the so-called ^5^ **F** matrix (called “FTDM matrix”) of ref 49 in this work, the elements of which are given by eq 3.

FAB5=∑μ∈A∑ω∈B(S1/2P[AO]S1/2)μω2∑μ∈complex∑ω∈complex(S1/2P[AO]S1/2)μω2 3

*A* and *B* are the molecular fragments, and **S** is the atomic orbital overlap matrix, which can be directly obtained from matrix **c** containing the molecular orbital coefficients (with one column per molecular orbital) (eq 4):^48^

$$S=(c^{T}{)}^{-1}c^{-1}$$ 4

The sum of all ^5^ *F*_*AB*_ elements is equal to 1. From a practical standpoint, the ^5^ **F** matrix can be efficiently computed and provides easy-to-interpret numerical values if the system is divided into chemically reasonable fragments. The choice of suitable fragments, in turn, depends on the scientific question to be answered as well as, to some extent, on the chemical intuition of the user.

The required **P** ^[AO]^ and **S** matrices for this study were obtained from Multiwfn 3.8 in the case of DFT, while the ^5^ **F** matrices themselves were calculated using an in-house python script. Since *C*_ov_ coefficients are not naturally printed out by ORCA for TD-DFT jobs, we recovered the coefficients from the CI vectors stored in the .cis binary using an in-house python script, with the code partially adapted from Theodore.^97^ In the case of TD-DFTB, a mixture of in-house written python and C++ scripts were employed for the entire FTDM workflow. For DFT and CIS, only *C*_ov_ coefficients larger than 10^–4^ were included in the FTDM analyses.

## 3 Results and Discussion

### 3.1 Exemplary Excited-State Analysis for Upright *trans*-AB on Au_25_

We want to begin by discussing a representative example system to illustrate the excited-state characterization outlined in the last section. Figure 2 summarizes the excited-state analysis results for upright *trans*-AB with two CH_2_ spacer units on Au_25_ employing TD-CAM-B3LYP/def2-TZVP. The highest absorption values in the UV/vis absorption spectrum (Figure 2a) are found slightly below 3.7 eV. A spectral comparison with the free *trans*-AB and gold+SCH_3_ systems in Figure S1a in the SI indicates that the absorption maximum overlaps with the ππ* absorption band of *trans*-AB. At lower excitation energies below 3 eV, only weak absorption is observed. It should however be noted that the UV/vis spectrum is functional-dependent. An overview of the computed absorption spectra for various computational methods is given in Figure S2 in the SI.

**Figure 2:**
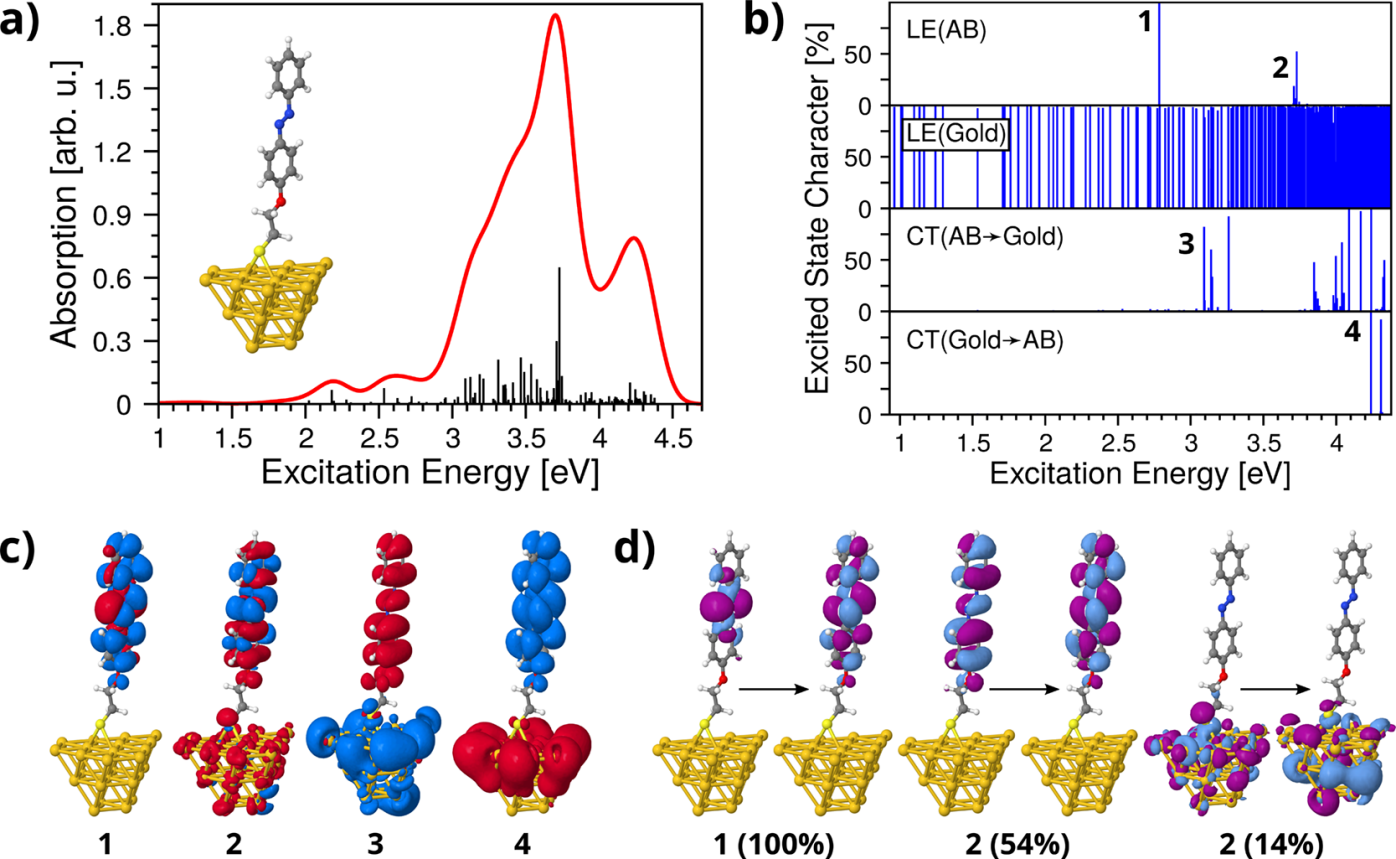
Excited-state analysis of upright *trans*-AB on Au_25_ at the TD-CAM-B3LYP/def2-TZVP level of theory. (a) UV/vis absorption spectrum of the hybrid AB–gold system. Black sticks correspond to the individual electronic transitions. (b) Full FTDM analysis plot including all LEs of and CT excitations between the AB+spacer and Au_25_+S fragments. (c) CDDs of four selected excited states from (b). Increased electron densities are shown in blue, and reduced electron densities are shown in red. (d) NTOs for the AB local excited states in (b). Contributions of individual hole-particle pairs are given in percent in brackets.

To characterize all computed excited states, the FTDM analysis showing all LEs of and CT excitations between the *trans*-AB+alkyl spacer and the Au_25_ cluster is given in Figure 2b. We generally include the binding S atom as a part of the gold cluster in the FTDM analysis due to many partial S↔gold CT interactions (see Figure S1b in the SI), which result from a strong orbital overlap. Most of the excited states in Figure 2b are LEs of the gold cluster owing to its metallic character. LEs of the *trans*-AB are observed at 2.78 eV and around 3.73 eV, which match the S_1_ and S_2_ excitation energies of the free *trans*-AB+alkyl spacer in vacuum [*E*(S_1_/S_2_) = 2.78/3.74 eV]. The first AB excited state (nπ*) at 2.78 eV is fully localized on the molecule [100% LE­(AB)]. The second excited state (ππ*) is resonant to close-lying local gold excitations, given by fractional LE contributions of both the AB (53%) and the gold cluster (47%). Furthermore, multiple AB → gold CT excitations are observed above 3 eV, while gold → AB CT excitations are only sparsely found above 4 eV. The preference toward AB → gold CT is qualitatively in line with earlier experimental reports^16–18^ suggesting hole transfer-accelerated AB isomerization near gold nanoparticles, albeit in the electronic ground state. Moreover, hole transfer leading to formation of AB radical cations is also favored due to the substitution pattern involving a donating ether residue.^98^ The calculated CT excitation energies are however strongly method-dependent as shown in Figure S3 in the SI. Moreover, it should be noted that CT excitations are in general challenging to describe using TD-DFT,^99^ even if long-range corrected functionals such as CAM-B3LYP are employed. We thus primarily focus on the behavior of local AB excited states at the metal interface in this study.

To validate the FTDM analysis results, CDDs and NTOs of selected excited states are shown in Figure 2c,d. Both LEs of the AB and the two CT excitations are well represented by the CDDs in Figure 2c. Additionally, the NTOs in Figure 2d confirm the primary AB LE to be of nπ* character, while the second AB LE corresponds to the AB ππ* state as expected.^29^ The full localization of the nπ* state on the *trans*-AB as observed in the FTDM analysis results from the near-zero transition dipole moment of the nπ* excited state. Meanwhile, the high-transition dipole moment of the *trans*-AB ππ* state leads to resonant coexcitation with local gold states in the FTDM analysis as discussed earlier. We will later show that these qualitative observations typically hold true for other tested functionals, basis sets, and gold clusters.

### 3.2 Variation of the Alkyl Spacer Length for Upright *trans*-AB on Au_25_

Using our FTDM analysis procedure, we now want to focus on the influence of the alkyl spacer length on the local AB excited states for upright *trans*-AB on Au_25_. Figure 3a shows the FTDM elements corresponding to local AB excitation [LE­(AB)] around the ππ* excitation energy (TD-CAM-B3LYP) for four different alkyl spacers. The structures of the four AB–gold hybrid systems are shown in the top right of Figure 1. Although the previously mentioned resonance effects lead to a splitting of the ππ* excitation into multiple close-lying excited states with fractional LE­(AB) values, the ππ* state becomes gradually localized on the AB with increasing AB–gold distance. The small visible shifts of ∼10 meV for longer spacers compared to two CH_2_ units are also observed for the corresponding free *trans*-AB molecules in vacuum. We thus attribute these shifts to electronic interactions between the AB chromophore and the alkyl spacer. In contrast, the AB nπ* state is always fully localized on the molecule for TD-CAM-B3LYP and most other tested density functionals, irrespective of the alkyl spacer length. In rare cases, however, resonant coexcitation of the AB and gold cluster can also be observed for the nπ* state at short AB–gold distances. A corresponding example at the TD-PBE level is shown in Figure S4a in the SI.

**Figure 3:**
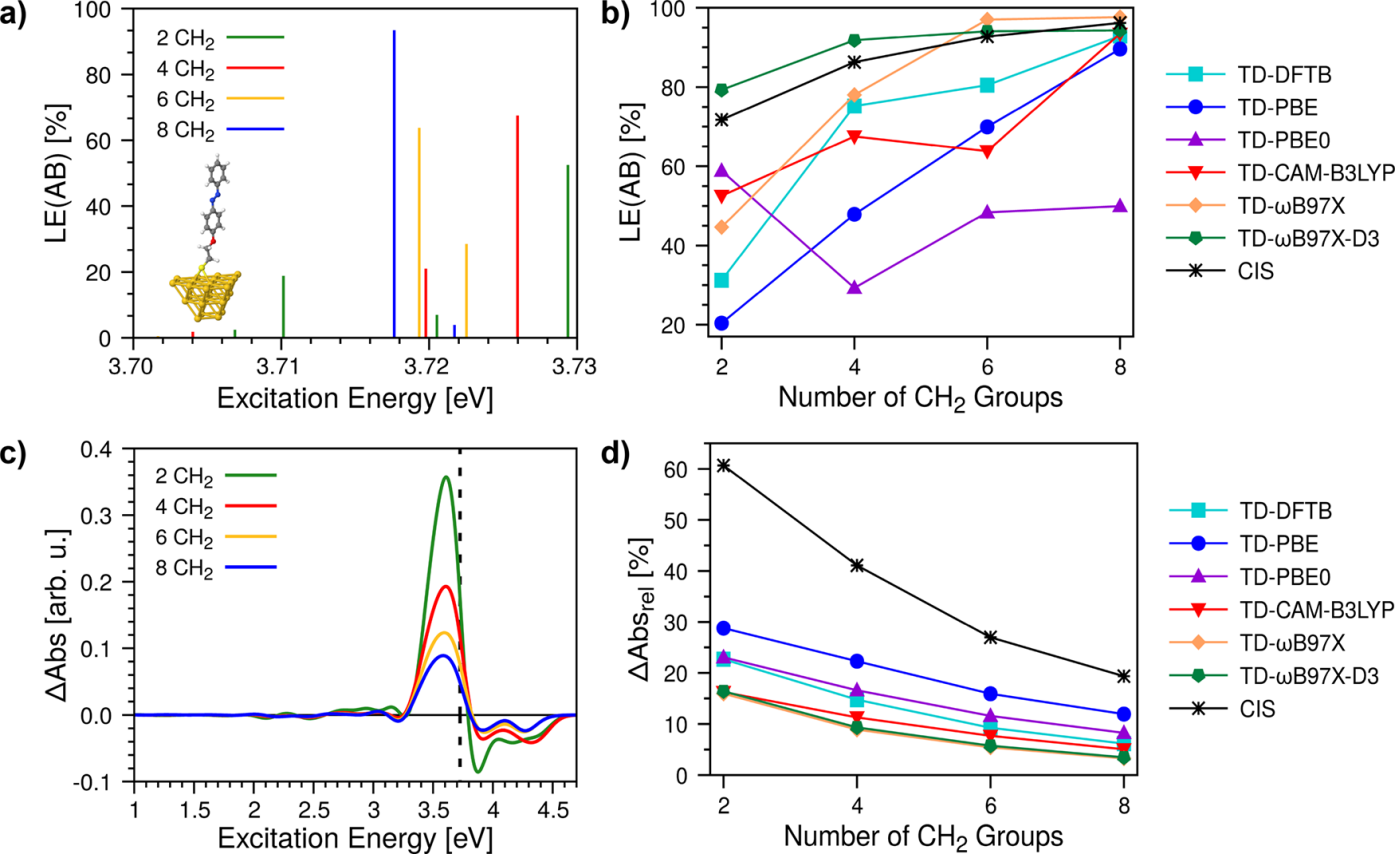
Excited-state analysis of upright *trans*-AB on Au_25_ as a function of the alkyl spacer length. (a) FTDM analysis of the AB ππ* state at the TD-CAM-B3LYP/def2-TZVP level of theory. (b) Localization of the AB ππ* state on the molecular part of the hybrid systems as a function of the alkyl spacer length for different computational methods. (c) Absorption difference spectra at the TD-CAM-B3LYP/def2-TZVP level of theory (see eq 5 and related explanations on the calculation of ΔAbs). The dashed black line shows the excitation energy of the AB ππ* state as highlighted in (a). (d) Relative absorption increase of the hybrid systems at the AB ππ* excitation energy for different computational methods.

For a more systematic analysis of the AB ππ* localization, the corresponding LE­(AB) values from the FTDM analyses using TD-DFT (with various functionals), TD-DFTB, and CIS are shown in Figure 3b as a function of the alkyl spacer length. A related plot within the TDA is given in Figure S4b in the SI. The largest LE­(AB) elements attributed to the AB ππ* state were used for all data points since the ππ* excitation is split into multiple excited states due to resonant AB–gold coexcitation (Figure 3a). At short AB–gold distances, the degree of localization of the AB ππ* state on the molecular part visibly depends on the computational method, ranging from ∼20% (TD-PBE, TDA-PBE0) to ∼80% (TD-ωB97X-D3) LE­(AB) for two CH_2_ units. For eight CH_2_ spacer units, the ππ* state becomes over 90% localized on the AB chromophore for most tested methods. Qualitatively, an increasing degree of localization of the AB ππ* state with increasing spacer length is obtained for all tested methods except for TD-PBE0. We will later show that the quantitative values of LE­(AB) also depend on other computational parameters, most notably on the form/size of the gold cluster model.

To investigate the influence of the resonant AB–gold excitations on the UV/vis absorption, we calculated the absorption difference spectra (ΔAbs) based on the following equation:

$$\Delta \mathrm{Abs}=\mathrm{Abs}(\mathrm{AB}-\mathrm{Gold})-\mathrm{Abs}({\mathrm{CH}}_{3}S-\mathrm{Gold})-\mathrm{Abs}(\mathrm{AB})$$ 5

Here, at any given excitation energy, Abs­(AB–Gold), Abs­(AB), and Abs­(CH_3_S–Gold) are the absorption values of the hybrid AB–gold system, of the isolated AB in vacuum, as well as of the isolated gold cluster with a chemisorbed SCH_3_ group at the AB binding position, respectively. The SCH_3_ group was added to account for the strong gold–sulfur interactions mentioned earlier, which influence the absorption spectrum of the isolated gold cluster (see Figure S1a in the SI). If no electronic interactions occurred between the AB and the gold surface in the hybrid system, the calculated absorption spectrum should be similar to the sum of the individual absorption spectra of the isolated parts, and ΔAbs should in turn be small. As shown in Figure 3c for TD-CAM-B3LYP, ΔAbs is indeed close to 0 up to 3.3 eV. At higher excitation energies, ΔAbs rapidly increases, reaching its maximum value at ∼3.6 eV. The positive peak of ΔAbs indicates increased absorption of the hybrid system in the vicinity of the AB ππ* state relative to the isolated fragments. We note that the relative position of the maximum of ΔAbs and the AB ππ* excitation energy is method-dependent: The ππ* state is blue-shifted by about 0.1 eV in the case of TD-CAM-B3LYP (see the dashed line in Figure 3c) while lying on the top of the maximum for ΔAbs in the case of TD-PBE (see Figure S4c). Furthermore, the maximum value of ΔAbs depends on the length of the alkyl spacer and hence on the AB–gold distance.

To quantitatively compare the behavior of ΔAbs among different computational methods, we further define the dimensionless quantity ΔAbs_rel_ as

$$\Delta {\mathrm{Abs}}_{\mathrm{rel}}=\frac{\Delta \mathrm{Abs}}{\mathrm{Abs}(\pi \pi_{\mathrm{AB}}^{*})}\times 100\%$$ 6

with Abs­(ππ_AB_^*^) given as the absorption value of the free AB molecule including the linker and the spacer in vacuum at the ππ* excitation energy. Figure 3d shows the calculated values for ΔAbs_rel_ at the excitation energy of the AB ππ* state (on Au_25_) employing TD-DFT, TD-DFTB, and CIS as a function of the alkyl spacer length. Results within the TDA are summarized in Figure S4d. ΔAbs_rel_ systematically decreases with increasing spacer length for all tested methods, in line with the increasing localization of the AB ππ* state on the molecule (Figure 3b). For the shortest alkyl spacer, ΔAbs_rel_ varies between 10 and 30% except for ∼60% using CIS. In contrast to the behavior of LE­(AB) in Figure 3b, ΔAbs_rel_ shows a systematic dependence on the underlying density functional approximation: The GGA functional PBE gives the second largest values of ΔAbs_rel_ after CIS, which decrease upon introducing a fixed amount of exact exchange in the global hybrid functional PBE0. ΔAbs_rel_ further decreases for the three range-separated hybrids, while TD-DFTB lies between PBE0 and CAM-B3LYP. Interestingly, the DFT results systematically suggest a decrease of ΔAbs_rel_ by increasing the amount of exact exchange of the functional, while CIS instead gives the largest ΔAbs_rel_ values of all tested methods. We later show, however, that CIS even provides qualitatively different results in certain cases and should therefore be carefully evaluated. Furthermore, we note that, in general, both the calculated excitation energies and transition dipole moments have their own method-dependent, yet independent errors. Nevertheless, we rely on the oscillator strengths (which depend on both quantities) as a physically sound measure for absorption when calculating ΔAbs_rel_ values in the present work.

### 3.3 Influence of the Gold Cluster Form/Size and Other Computational Parameters on LE­(AB) and ΔAbs_rel_

To summarize the main conclusions for upright *trans*-AB on Au_25_ up to now, we have found the following: (i) the excitation energies of the AB excited states remain virtually unaffected upon chemisorption of the AB on small gold clusters, even if relatively short alkyl spacers are used. (ii) The *trans*-AB nπ* state is fully localized on the molecule for most tested methods due to its near-zero transition dipole moment, and (iii) Resonant coexcitation of the AB ππ* excited state and local gold states is observed, and the ππ* excitation is split into multiple excited states and becomes increasingly localized on the molecule with increasing AB–gold distance. Additionally, enhanced UV/vis absorption is observed around the ππ* excitation, the effect of which decreases for longer alkyl spacers. While all of these qualitative trends hold true for most tested computational methods, the quantitative results are visibly affected by, e.g., the chosen density functional. We now want to use the two quantities LE­(AB) and ΔAbs_rel_, both for the AB ππ* excitation, to investigate the quantitative influence of other computational parameters. We will focus on upright *trans*-AB with the shortest alkyl spacer consisting of two CH_2_ units in the next part since the AB–gold interactions become stronger with decreasing distance.

Figure 4a summarizes the calculated values of LE­(AB) and ΔAbs_rel_ for a set of eight different gold clusters at the TD-CAM-B3LYP level. Corresponding results within the TDA are shown in Figure S5 in the SI. Most of the utilized cluster models were chosen based on the general criteria outlined in the methods section. The structure of the two-layered (2L) Au_25_ cluster was adapted from the original publication on the auorg-1-1 parameter set,^89^ which was also used for the TD-DFTB calculations in this study. The Au_24_ cluster was chosen as an example to include the hydrogen atom from the deprotonated thiolate group of the AB while keeping an overall closed-shell nature of the system.

**Figure 4:**
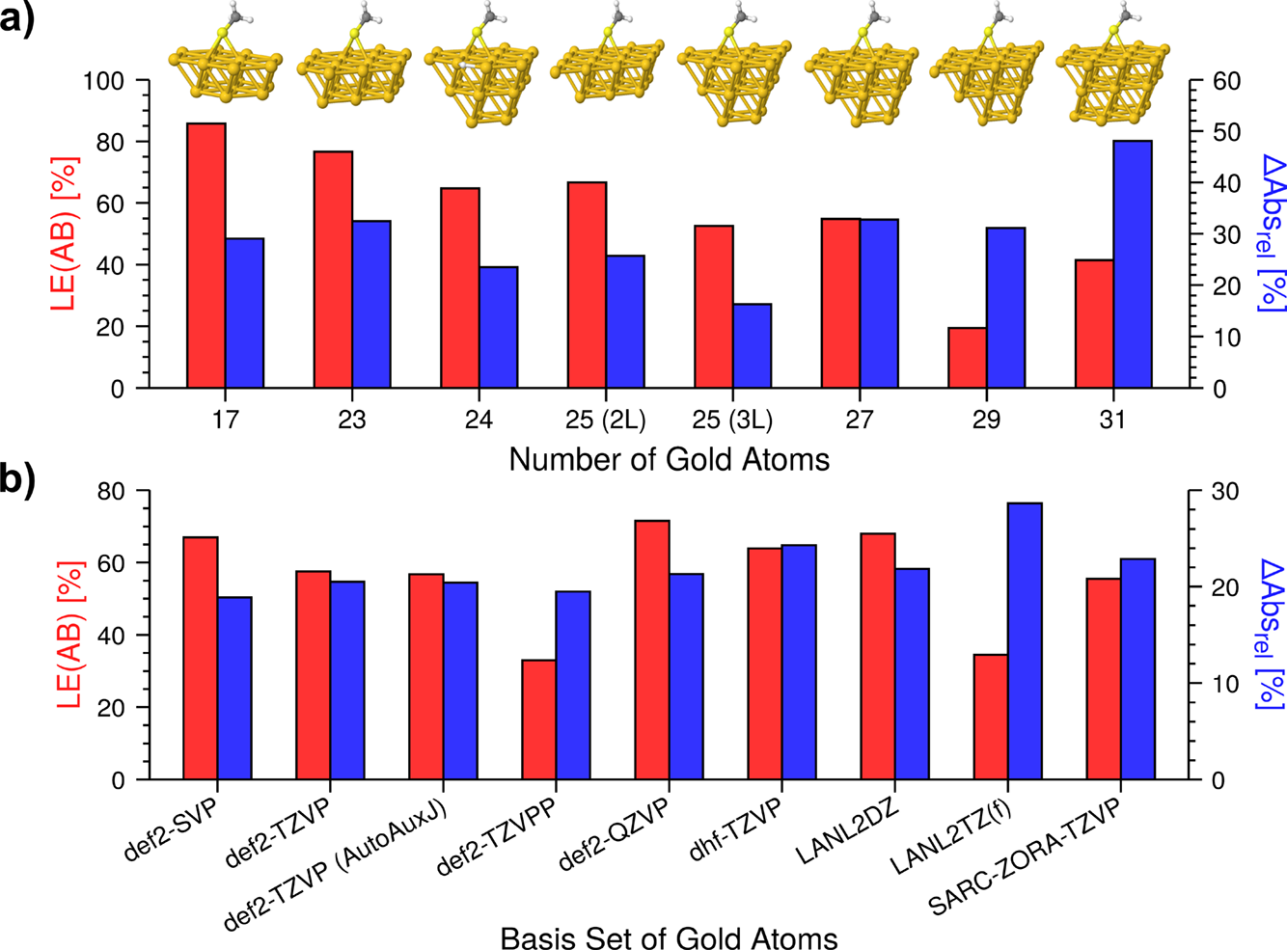
Influence of the gold cluster form/size and chosen basis set on the excited states of upright *trans*-AB with two CH_2_ spacer units on gold. The localization of the AB ππ* state is shown in red, and the relative absorption increase at the AB ππ* excitation energy is shown in blue. (a) Results for different gold clusters at the TD-CAM-B3LYP/def2-TZVP level. The utilized cluster models are shown above the plot, and the SCH_3_ groups show the binding positions of the AB molecule. (b) Results employing TDA-CAM-B3LYP with different basis sets on Au_25_ (3L). In the case of def2 and dhf basis sets, the same basis set as for the gold atoms was used for the molecular part. The two LANL and the SARC-ZORA-TZVP basis sets were paired with the 6-31G* and ZORA-def2-TZVP basis sets for the molecular part, respectively.

Although the qualitative trends reported thus far were found for all of the tested cluster models, the quantitative results in Figure 4a are strongly dependent on the chosen gold cluster. The resonant coexcitation of the AB ππ* state generally depends on the number of local gold states in close proximity and their transition dipole moments. Increasing the number of local gold states leads to an increased splitting of the ππ* excitation, affecting both the LE­(AB) and ΔAbs_rel_ values in Figure 4a. The local gold excited states in turn strongly depend on the form and size of the cluster model. To visualize this dependence, the UV/vis absorption spectra of the eight cluster models at both the TD-DFT level and within the TDA are summarized in Figure S6 in the SI. The increased splitting of the AB ππ* excitation for a higher number of close-lying local gold states becomes evident when comparing the FTDM analyses for upright *trans*-AB on Au_25_ in Figure 3a and the much larger Au_73_ cluster in Figure S7 in the SI. Moreover, the strong splitting on Au_73_ leads to a redshift of the ππ* excitation by about 0.1 eV compared to the free AB molecule, similar to what one would expect, e.g., in J-type molecular aggregates.^100^ We further address this point later on when discussing the even larger Au_201_ cluster model.

Other computational parameters influencing the calculated excited states of the AB–gold hybrid systems are expected to also quantitatively influence the results for LE­(AB) and ΔAbs_rel_. Figure 4b summarizes the basis set dependence of the calculated quantities for upright *trans*-AB on Au_25_ (3L) at the TDA-CAM-B3LYP level. On average, many of the tested basis sets give similar results for LE­(AB) and ΔAbs_rel_ compared to the much larger deviations between different gold clusters in Figure 4a. Def2-TZVPP and LANL2TZ­(f) most notably affect the quantitative results in the present example, although the relative performance of different basis sets may potentially change when employing a different cluster model. We also tested the inclusion of the upper gold cluster layer in the initial geometry optimization of the AB–gold system to approximately include reconstruction effects of the gold surface, which we have neglected until now. Figure S8 in the SI compares the computed LE­(AB) and ΔAbs_rel_ values as a function of the alkyl spacer length for upright *trans*-AB on Au_25_ with an unfixed first layer compared to the results for the fully frozen cluster model at the TD-CAM-B3LYP/def2-TZVP level. While unfixing the upper gold layer quantitatively affects the results somewhat, the qualitative trends for LE­(AB) and ΔAbs_rel_ are unaffected. In this regard, we again emphasize that the main parameter governing the quantitative results in all our examples thus far is the form/size of the underlying cluster model.

### 3.4 Directly Bound *trans*-AB on Gold

Before focusing on the results for other AB isomers, we want to further discuss the role of the alkyl spacer separating the AB from the gold surface. To this end, we investigated the excited states of *trans*-AB directly linked to the gold cluster via a sulfur atom in Figure 5. This case is especially relevant for other commonly probed molecules on plasmonic nanoparticles such as the widely studied 4-NTP,^11–15^ which are not typically separated from the metal surface by an alkyl spacer.

**Figure 5:**
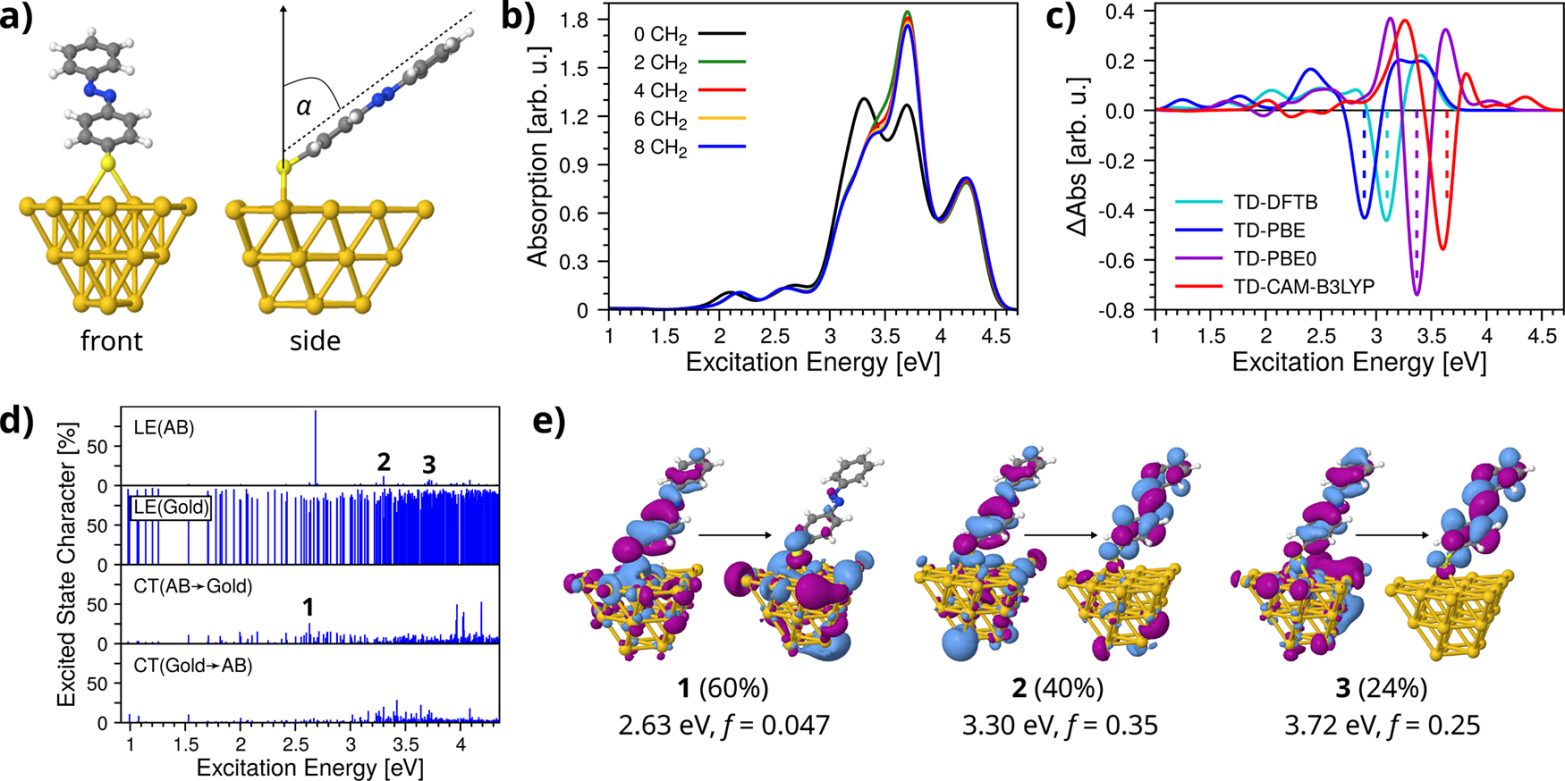
Excited-state analysis of *trans*-AB directly bound to Au_25_. (a) Front and side views of the TPSS/def2-TZVP optimized geometry of the AB–gold model. (b) UV/vis absorption spectrum of the model in (a) compared to the spectra for upright *trans*-AB on Au_25_ with different spacer lengths at the TD-CAM-B3LYP/def2-TZVP level. (c) Absorption difference spectra of (a) for four different computational methods. Colored dashed lines show the position of the ππ* state of the free AB thiol. (d) Full FTDM analysis of (a) using TD-CAM-B3LYP/def2-TZVP. (e) NTOs for three selected states from (d) including excitation energies and oscillator strengths *f*. Contributions of individual hole-particle pairs are given in percent in brackets.

The optimized geometry of the directly bound AB on Au_25_ is shown in Figure 5a. The tilt angle between the surface normal of the cluster and the molecular plane is α = 56° for TPSS/def2-TZVP. Hekele et al.^101^ recently reported a slightly smaller angle of 53° for a medium coverage of the structurally similar 4-NTP on Au(111) employing periodic DFT calculations at the PBE level. The direct AB–gold connection visibly affects the UV/vis absorption spectrum of the hybrid system in Figure 5b compared to the systems including an alkyl spacer. Around the ππ* excitation energy of the free AB thiol, the absorption strongly decreases in Figure 5c (negative ΔAbs values), while increased absorption is observed at both higher and lower excitation energies for different computational methods. Indeed, the FTDM analysis in Figure 5d only shows the AB nπ* state at 2.68 eV as primarily localized on the molecule, while a clear peak corresponding to the AB ππ* state (3.64 eV for TD-CAM-B3LYP) is missing. The primary reason is a direct hybridization of the AB molecular orbitals with orbitals from the gold cluster as shown by the NTOs for three example states in Figure 5e. We note that this effect differs from the resonant coexcitation including an alkyl spacer as discussed earlier: In the present case, delocalized hybrid states consisting of local gold orbitals and π-type orbitals of the AB are excited, while the ππ* state is fully located on the molecule but resonant to local gold excitations in the case of resonant coexcitation. As a consequence of the hybridization, local AB excitations with small LE­(AB) values are visible at various excitation energies in Figure 5d. Furthermore, due to the inclusion of π-type molecular orbitals of the AB, these states are typically somewhat bright with oscillator strengths of *f* ≥ 0.05. In sight of our earlier results, the present findings underline the capability of even short alkyl spacers (e.g., consisting of two CH_2_ units) to electronically decouple the AB molecular orbitals from the gold cluster.

### 3.5 Excited-State Analyses for Other AB Isomers on Au_25_

Excluding the case of directly bound *trans*-AB on Au_25_, all our results thus far focus exclusively on a perpendicular orientation of *trans*-AB molecules relative to the gold surface. Under experimental conditions, the structure relationship between the substrates and the metal surface is generally more complex: (i) the orientation of AB molecules relative to the surface is coverage dependent. At low surface coverage, AB molecules may, e.g., adopt a more horizontal (“flat”) orientation as substrate–surface interactions become increasingly important relative to substrate–substrate interactions. The situation becomes even more complex when long and flexible spacer units with many freely rotatable bonds are involved, which may increase the conformational variety of the surface-bound adsorbates. Such a conformational analysis, e.g., for *trans*-AB with eight CH_2_ spacer units, is however beyond the scope of the present study. (ii) *Trans*-AB molecules can undergo a light-induced isomerization reaction to the energetically less favored *cis*-form, thus changing the molecular structure. (iii) The orientation of the surface-bound molecules is experimentally influenced by nuclear motion, while our calculations assume an idealized static case. We aim to address the latter point in a separate computational study and focus on points (i) and (ii) in the present work.

Figure 6 summarizes the excited-state analysis results for flat *trans*-AB and upright *cis*-AB on Au_25_ focusing on the AB ππ* excitation employing TD-DFT, TD-DFTB, and CIS. Results within the TDA as well as for flat *cis*-AB are shown in Figures S9 and S10, respectively. The structures of the three additional isomers for all four spacer lengths are summarized in Figure S11 in the SI. For flat *trans*-AB, the transition dipole moments of the AB excited states are oriented differently relative to the metal surface in comparison to upright *trans*-AB. Furthermore, the AB–gold distance is less affected by changing the length of the alkyl spacer due to the sideways orientation of the molecule. As a result, the AB ππ* excitation in Figures 6b and S9b becomes less strongly localized on the AB for longer alkyl spacers compared to upright *trans*-AB. In the present example, the LE­(AB) values still vary between 40 and 90% for eight CH_2_ units employing different computational methods, while values of ∼90% were obtained for a majority of the methods in Figure 3b. Moreover, contrary to the expected systematic decrease of ΔAbs_rel_ with increasing AB–gold distance, ΔAbs_rel_ increases from two to four CH_2_ units for some of the tested methods in Figures 6c and S9c. The main reason is the simultaneous appearance of both resonant coexcitation and hybridization effects for flat *trans*-AB with two CH_2_ units due to the very small distance to the gold surface. The observed hybridization in turn leads to smaller/negative ΔAbs_rel_ values at the ππ* excitation energy of the free AB molecule, as previously discussed for directly bound *trans*-AB on Au_25_ in Figure 5.

**Figure 6:**
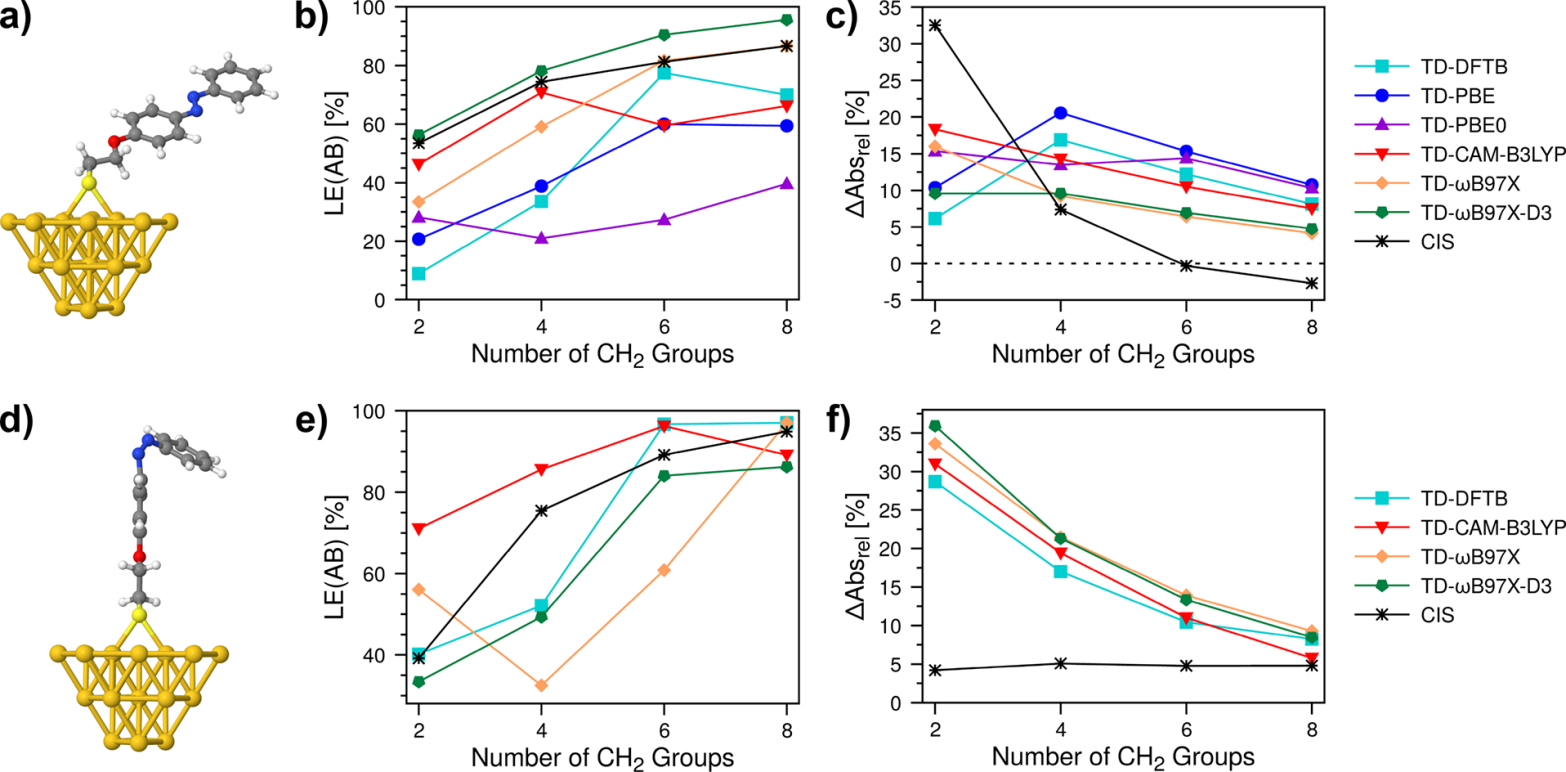
Excited-state analysis of other AB isomers on Au_25_ employing different computational methods. (a–c) Front view, localization of the AB ππ* excitation, and relative absorption increase at the ππ* excitation energy for flat *trans*-AB. (d–f) Results for upright *cis*-AB.

In comparison to upright *trans*-AB, isomerization to *cis*-AB influences the nπ* and ππ* excitation energies, leading to a redshift of the nπ* state by about 0.2 eV and a strong blueshift of the ππ* state by about 0.6 eV for TD-CAM-B3LYP. Additionally, the oscillator strength of the ππ* state strongly decreases for free *cis*-AB (*f* = 1.0 for *trans*- and *f* = 0.35 for *cis*-AB with two CH_2_ units at the TD-CAM-B3LYP level), while the nπ* state in turn becomes somewhat bright (*f* = 0.045 for *cis*-AB). Resonant coexcitation between the AB nπ* state and the local gold states is thus more frequently observed for upright *cis*-AB on Au_25_ at short spacer lengths. Two corresponding examples are shown in Figure S12 in the SI. The LE­(AB) and ΔAbs_rel_ values in Figures 6e,f and S9e,f for the ππ* excitation of upright *cis*-AB on Au_25_ follow the same qualitative trends with increasing AB–gold distance as previously discussed for upright *trans*-AB in Figure 3. Quantitatively, the values of ΔAbs_rel_ vary between ∼30% (TD-DFTB) and ∼35% (TD-ωB97X-D3) for upright *cis*-AB considering two CH_2_ units when excluding CIS and are thus somewhat larger compared to our earlier results for upright *trans*-AB on Au_25_. We do however emphasize that the quantitative differences are not necessarily a result of a stronger interaction between *cis*-AB and the gold cluster: Since isomerization to *cis*-AB leads to a blueshift of the ππ* state, the number of local gold states around the ππ* excitation energy also changes. The number of close-lying local gold states and their transition dipole moments in turn determine possible resonance effects and thus quantitatively affect both LE­(AB) and ΔAbs_rel_. We have discussed this effect in a similar way in Figure 4a by varying the form and size of the cluster geometry, which influences the number of close-lying local gold states at a given excitation energy.

The results for flat *cis*-AB on Au_25_ in Figure S10 follow the qualitative trends previously discussed for flat *trans*-AB. The hybridization effect in the case of two CH_2_ units is more pronounced for flat *cis*-AB and leads to initially negative ΔAbs_rel_ values in many cases. Moreover, we note that the results for ΔAbs_rel_ follow qualitatively different trends for *cis*-AB in both orientations relative to the gold surface when employing CIS compared to TD­(A)-DFT and TD-DFTB: For CIS in the upright case, ΔAbs_rel_ remains almost constant, independent of the AB–gold distance (Figure 6f) while systematically increasing for longer alkyl spacers in the case of flat *cis*-AB (Figure S10c). In contrast, the CIS results are, at least qualitatively, in line with TD-DFT for the *trans*-ABs in Figures 3d and 6c. We therefore argue that the reliability of CIS for *cis*-AB on gold generally remains to be questioned, and the corresponding results should hence be carefully evaluated. A more in-depth study concerning the performance of CIS for hybrid AB–gold systems is however beyond the scope of this work, which primarily focuses on DFT and DFTB methods.

### 3.6 Exciton Formation in *trans*-AB Dimers on Au_64_

Up to now, we have only focused on the excited states of single AB molecules at the metal interface. However, neighboring AB molecules on the surface may also form molecular aggregates due to π–π stacking interactions as shown in Figure 7a. It is well-known that *trans*-AB aggregates in the gas phase can give rise to the formation of molecular excitons.^49–52,102,103^ Such excitonic states in *trans*-AB dimers and their behavior at the metal interface can also be efficiently described using our FTDM analysis procedure, which we want to demonstrate in the following.

**Figure 7:**
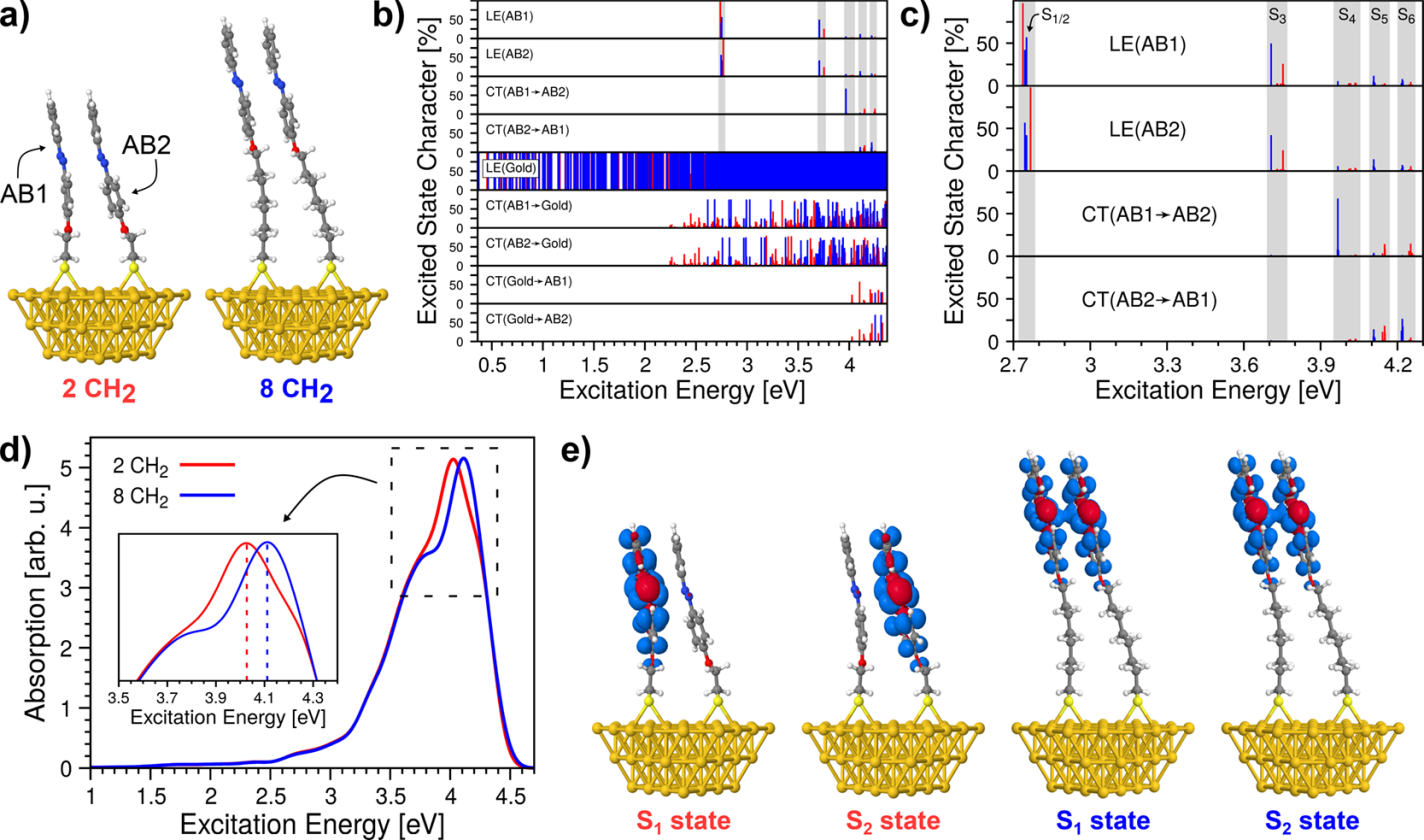
Excited-state analysis of *trans*-AB dimers with two and eight CH_2_ units on Au_64_ at the TDA-CAM-B3LYP/def2-SVP level. (a) TPSS/def2-SVP structures of the two AB-dimer hybrid models. (b) Full three fragment FTDM analyses of the structures from (a) considering the two *trans*-ABs as individual fragments and the Au_64_ cluster including the binding sulfur atoms as the third fragment. (c) LEs of and CT contributions between the two ABs from (b). Molecular excited states are highlighted in gray. The excited-state labels were adapted from the corresponding results for the free *trans*-AB dimers in vacuum (see Figure S14a in the SI). (d) UV/vis absorption spectra of the systems in (a). (e) CDD plots of the first two molecular excited states marked in (c), with increased electron densities shown in blue and reduced electron densities shown in red. Results for the model with two CH_2_ units are shown in red and with eight CH_2_ units are shown in blue across the figure.

Figure 7 summarizes the excited-state analysis of *trans*-AB dimers with two and eight CH_2_ units on Au_64_ employing TDA-CAM-B3LYP/def2-SVP. Results at the TD-DFTB level are summarized in Figure S13 in the SI. The FTDM analyses for the free *trans*-AB dimers excluding the metal surface at both levels of theory are shown in Figure S14. A comparison of the UV/vis absorption spectra of the free *trans*-AB dimers and the corresponding monomers is given in Figure S15, demonstrating a blueshift upon dimerization. For the AB dimers on Au_64_, we performed three component FTDM analyses with the two surface-bound ABs treated as individual fragments, while the third fragment consists of the Au_64_ cluster including the binding sulfur atoms. At first glance, the full FTDM plot in Figure 7b is qualitatively similar to our earlier result for the upright *trans*-AB monomer on Au_25_ in Figure 2b. The main difference is the number of (partially) local AB excited states in the given energy range: While only two states (nπ* and ππ*) are observed for the monomer, six states for each of the two spacers are visible in case of the AB dimers for TDA-CAM-B3LYP when including both LEs of and CT between the two monomers. The first two local AB excited states in Figure 7c are observed between 2.7 and 2.8 eV, which matches the nπ* excitation energy of a single isolated *trans*-AB molecule at the same level of theory (2.78 eV for two CH_2_ units with the def2-SVP basis set). In the case of the short alkyl spacer, the two states are fully localized on one respective monomer with an energy difference of ∼30 meV. In contrast, delocalized dimeric transitions are observed for the long alkyl spacer, which are commonly known as Frenkel excitons.^48^ Due to the near-zero transition dipole moment of the *trans*-AB nπ* state, the nπ* exciton coupling is very weak, and Frenkel exciton formation, hence, requires near perfect stacking of individual AB monomers with almost identical geometries.^104^ Indeed, the spacer consisting of eight CH_2_ units in Figure 7a provides sufficient conformational freedom for the aggregation of the two monomers in an offset stacking pattern, similar to the optimized geometry of a free *trans*-AB dimer in vacuum.^49^ The short alkyl spacer on the other hand considerably restricts the aggregation possibilities of the monomers at the gold surface. As a result, one monomer (AB2) adopts a slightly twisted nonplanar geometry to enable the energetically beneficial π–π stacking of the upper aromatic rings, thus forming a distorted *trans*-AB dimer. The slight twisting leads to a small mismatch of the nπ* excitation energies of the two monomers and hence to the nπ* localization in Figure 7c. This effect is confirmed by the CDDs for the two excited states in Figure 7e, which clearly show localized/delocalized molecular excitations in the case of the short/long alkyl spacers, respectively. We further note that identical results are also obtained for the free AB dimers in Figure S14a, showing that the gold surface primarily has a geometrical influence on the AB dimers in the present case. Additionally, the transition dipole moments of the localized nπ* transitions in the distorted AB dimer with the short alkyl spacer are roughly 1 order of magnitude larger compared to the delocalized nπ* transitions in the respective dimer with the longer alkyl spacer.

The higher excited states of the AB dimers (S_3–6_) in Figure 7c are observed in a 0.4 eV range around the ππ* excitation energy of the free *trans*-AB monomer (4.06/4.04 eV for two/eight CH_2_ units with the def2-SVP basis set). The S_3–6_ states are dominated by a mixture of both local and CT excitations. We note that the ππ* excitons are delocalized for both spacer lengths, in line with stronger ππ* exciton coupling (than the nπ* one), which maintains delocalization despite geometrical differences of the monomers.^105^ In the case of the short alkyl spacer, the first bright excitation is the S_4_ state at 4.03 eV. The slight redshift relative to the free *trans*-AB monomer in vacuum results from resonance effects between the AB dimer excitation and local excited states of the gold cluster. In contrast, the S_5_ state at 4.11 eV has the largest transition dipole moment in the case of the long alkyl spacer. The eight CH_2_ AB dimers therefore show a small blueshift of the bright ππ* excitation relative to the monomer excited states as expected for H-type molecular aggregates within the Kasha exciton model.^100^ The small blueshift is clearly visible in the UV/vis absorption spectra of the AB dimers on Au_64_ in Figure 7d. Additionally, the bright states of the AB dimers are resonant to LEs within the gold cluster in Figure 7b,c, leading to a further splitting of the individual dimer excited states into multiple close-lying excitations. This effect is qualitatively identical to the resonant coexcitation previously discussed for the AB monomers on smaller gold clusters.

In comparison to TDA-CAM-B3LYP, TD-DFTB qualitatively reproduces the blueshift of the absorption maximum in the case of the longer alkyl spacer in Figure S13b. The behavior of individual excited states is however more complex in the case of TD-DFTB as the *trans*-AB dimers have over 20 excited states each in a range up to 3.7 eV (see Figures S13d and S14b in the SI). We therefore refrain from discussing all of the dimer excitations in detail and only focus on the energetically well separated nπ* excitation between 2.0 and 2.15 eV in Figure S13d. In contrast to TDA-CAM-B3LYP, the nπ* excitation is split into four excited states for each of the two alkyl spacers at the TD-DFTB level. In the case of the long spacer, two of the four states are local nπ* excitations, one localized on each respective monomer. The remaining two states are CT excitations from AB1 to AB2 and vice versa. For the short alkyl spacer, the separate LEs and CT excitations fuse into mixed excited states. Each monomer has two separate excited states showing simultaneous LE of that monomer and partial CT to the neighboring AB. Qualitatively identical trends are also observed for the *trans*-AB dimers in vacuum employing TD-DFTB in Figure S14b. While TD-DFTB thus also shows a general change in the dimer excitations between the two alkyl spacers, the qualitative nature of the dimeric transitions clearly differs from our earlier results at the TDA-CAM-B3LYP level. The main reason is the nature of the TD-DFTB method, a semiempirical time-dependent GGA-type approach, in contrast to CAM-B3LYP, which is a range-separated hybrid functional. We note that the influence of the underlying density functional approximation, albeit for global and range-separated hybrids, is also discussed in detail for *trans*-AB dimers in vacuum in ref 49.

### 3.7 FTDM+DFTB Excited-State Analysis for Upright *trans*-AB on Au_201_

Despite the qualitative differences in the case of the *trans*-AB dimers, the performance of TD-DFTB throughout this work is generally impressive: The method accurately captured all of the reported trends at the monomer level with near-DFT accuracy at a fraction of the cost of performing a full DFT calculation. TD-DFTB therefore enables atomistic simulations involving much larger metal clusters with many more excited states, which would otherwise remain beyond the scope of linear-response TD-DFT. Although experimental scales in the range of hundreds of thousands of metal atoms for actual nanoparticles remain challenging to achieve, metal clusters involving hundreds of atoms and tens of thousands of excited states are computationally accessible at the DFTB level.

To demonstrate the computational efficiency of our FTDM+DFTB workflow for large systems, we performed excited-state analyses for upright *trans*-AB with varying spacer lengths on Au_201_ in Figure 8. The cluster model was initially obtained from a Wulff construction and has an approximate diameter of 1.7 nm after full relaxation at the DFTB level. In contrast to the typical bridge-type binding of the AB on the gold surface for TPSS (see, e.g., Figure 1), DFTB instead predicts a monodentate binding position, as shown in Figure 8a. We note that test optimizations at the DFTB level involving the smaller gold clusters used throughout this study also resulted in similar on-top binding positions of the AB. Since bridge-type binding patterns of organic thiolates on Au(111) are commonly reported in the literature,^13,101,106^ the structural preference of DFTB is likely an effect of the semiempirical parameters taken from the auorg-1-1 parameter set.

**Figure 8:**
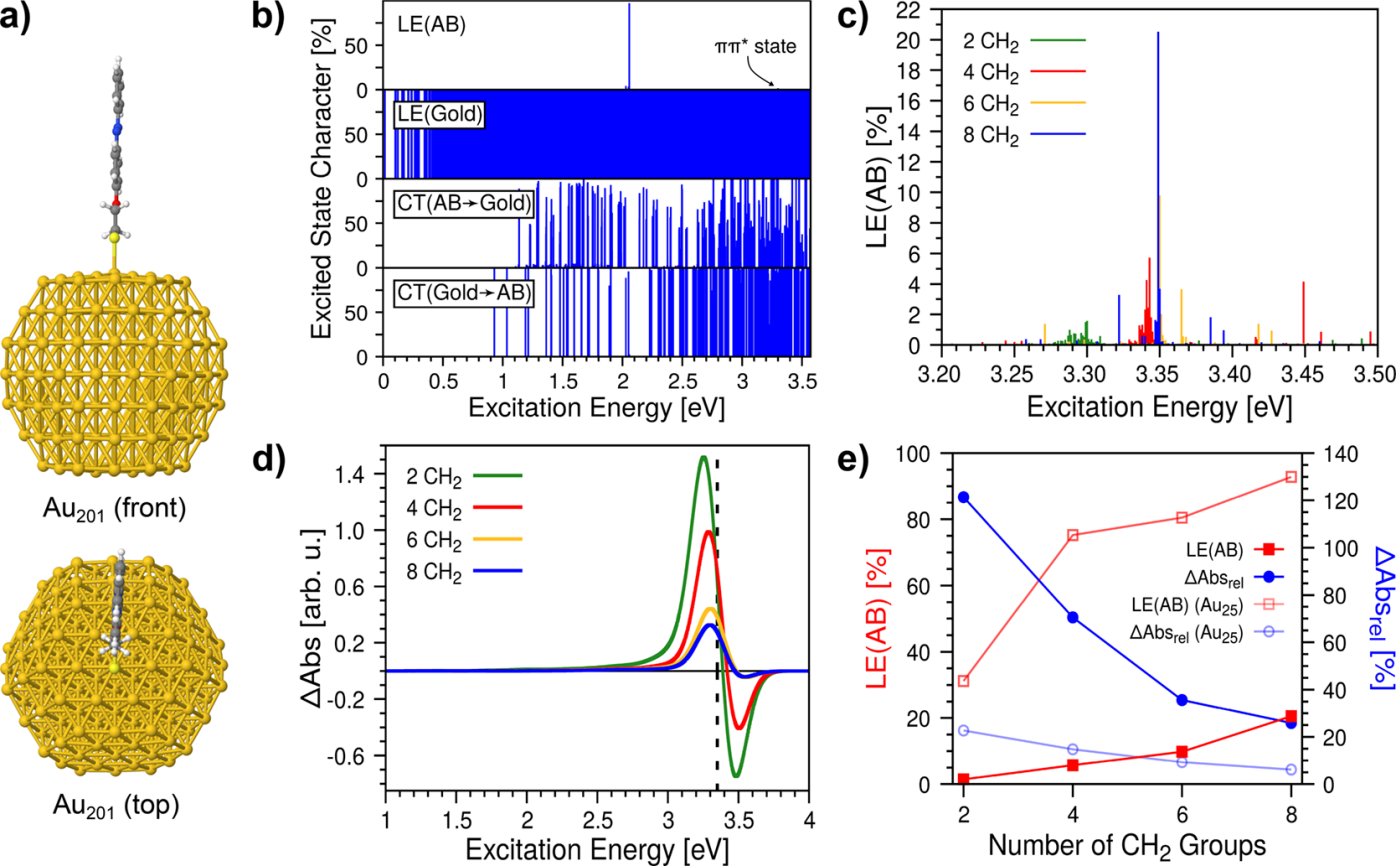
Excited-state analysis of upright *trans*-AB on Au_201_ employing TD-DFTB. (a) Front and top views of the fully optimized hybrid model with two CH_2_ units. (b) Full FTDM analysis of the model in (a). (c) FTDM analysis focusing on the local AB excitation at the ππ* excitation energy as a function of the alkyl spacer length. (d) Absorption difference spectra for different spacer lengths. The dashed black line shows the position of the AB ππ* state for an isolated *trans*-AB molecule in vacuum at the TD-DFTB level. (e) LE­(AB) (red) and ΔAbs_rel_ (blue) focusing on the AB ππ* excitation as a function of the alkyl spacer length. For easier comparison, the TD-DFTB results for upright *trans*-AB on Au_25_ from Figure 3 are also shown.

Figure 8b shows the full FTDM analysis for upright *trans*-AB with two CH_2_ units on Au_201_ including all 20,000 calculated states. Despite multiple LEs within the gold cluster per meV, the AB nπ* state at 2.06 eV is still clearly localized on the molecule. Furthermore, the nπ* excitation energy remains unaffected by the presence of the Au_201_ cluster relative to a free *trans*-AB molecule in vacuum at the same level of theory. In contrast, the AB ππ* excitation is resonant to close-lying local gold states and thus splits into many individual excited states with small LE­(AB) values in Figure 8b (<2%, see also Figure 8c). As shown in Figure 8c, the splitting of the ππ* excitation is strongly affected by the length of the alkyl spacer and visibly reduced with increasing AB–gold distance. Furthermore, the ππ* excitation is centered around 3.3 eV in the case of two CH_2_ units and thus red-shifted relative to the ππ* state at 3.43 eV for the corresponding isolated *trans*-AB in vacuum. A similar redshift was also obtained at the TDA-CAM-B3LYP level for upright *trans*-AB with two CH_2_ units on Au_73_ (Figure S7b). Since the ππ* excitation energy remains almost unchanged (shifts below 20 meV) for AB on smaller gold clusters such as Au_25_, we conclude that many close-lying resonant gold states are needed to induce a “visible” shift of the AB ππ* state at small AB–gold distances. We further emphasize that increasing the spacer length relative to two CH_2_ units leads to an additional small redshift of the AB ππ* state in vacuum. The ππ* excitation energy of 3.35 eV for *trans*-AB with eight CH_2_ units on Au_201_ is thus near identical to the computed value of 3.36 eV for the isolated AB including the linker and the spacer.

As shown in Figure 8d, the shift of the ππ* excitation energy is also visible in the absorption difference spectra and leads to a blueshift of the maximum of ΔAbs with increasing AB–gold distance. To account for this shift in the calculation of ΔAbs_rel_ for *trans*-AB on Au_201_, we used a slightly modified version of eq 6: the ΔAbs values were taken at the mean AB ππ* excitation energy of the hybrid systems as determined from the FTDM analyses, while the Abs­(ππ_AB_^*^) values were instead taken at the ππ* excitation energy of the respective free AB in vacuum. The resulting values for ΔAbs_rel_ and LE­(AB) for *trans*-AB on Au_201_ are summarized in Figure 8e in comparison to the TD-DFTB values on Au_25_ from Figure 3. Although the higher density of LEs and the larger number of gold atoms of the Au_201_ cluster strongly affect the quantitative results, the qualitative trends with increasing spacer length remain unchanged. We thus argue that the value of even small metal clusters in qualitative studies focusing on molecular excited states at the metal interface should not be understated if the results are carefully verified.

## 4 Conclusions

In the present study, we systematically investigated the excited states of covalently linked ABs on gold from a quantum chemical standpoint. The excited states of the hybrid metal–molecule systems were primarily computed within a linear-response TD-DFT formalism employing a wide variety of density functional approximations. The dense excited-state manifolds of the AB–gold systems were efficiently characterized via the FTDM approach by contracting the full TDM in atomic orbital basis onto individually defined fragments.

Our results show that the nπ* excited state remains almost unaffected by the chemisorption of *trans*-ABs in a perpendicular orientation on gold if the molecule is separated from the surface by a decoupling alkyl spacer. In contrast, the AB ππ* state is strongly resonant to close-lying local gold excitations. The observed resonance leads to a splitting of the ππ* excitation, increased UV/vis absorption around the ππ* excitation energy, and a visible red-shift of the ππ* state depending on the cluster size. With increasing AB–gold distance, the ππ* excitation becomes gradually decoupled from the local gold states and hence increasingly localized on the molecule. Qualitatively similar trends were also verified for other AB isomers, namely, horizontally oriented *trans*-AB as well as horizontally and perpendicularly oriented *cis*-AB. In contrast, directly attaching *trans*-AB molecules to the gold surface leads to the formation of delocalized hybrid states involving local orbitals of the gold cluster and π-type orbitals of the AB. Moreover, the newly formed hybrid states appear at various excitation energies and usually have nonzero transition dipole moments, making them potentially accessible to optical excitation. Similar, albeit weaker hybridization effects were also observed for horizontally oriented (“flat”) ABs with short alkyl spacers due to their very short distance to the gold surface. Furthermore, we characterized the excited states of π–π stacked *trans*-AB dimers, which are separated from the gold surface by alkyl spacers of different lengths. We found that sufficiently long spacers allow for a near-perfect stacking of neighboring ABs and lead to the formation of excitonic states at the metal interface. Meanwhile, short alkyl spacers significantly restrict the conformational freedom of the ABs, leading to the formation of distorted dimers, and thus, e.g., prevent the formation of nπ* Frenkel excitons.

Apart from molecular parameters, we further investigated the influence of various computational parameters on the simulated excited states. We were able to qualitatively verify all of the main trends across a variety of different density functionals, ranging from GGA to range-separated hybrids, many different basis sets, and various metal cluster models of different forms and sizes. Additionally, we found that semiempirical DFTB was capable of reproducing most of the present findings with near-DFT accuracy while greatly reducing the computational cost. TD-DFTB thus provides an efficient framework for tackling larger systems beyond the computational limitations of DFT. However, our systematic analysis also shows that quantitative results are strongly tied to the computational setup and should, thus, be critically evaluated and compared.

In the future, we aim to further broaden the understanding of covalently linked ABs at the metal interface by going beyond purely static models toward nonadiabatic dynamics of hybrid AB–gold systems. To do so, we will perform trajectory surface-hopping dynamics simulations at the TD-DFTB level as recently demonstrated for bare Au_20_ and Au_20_–CO systems in related computational studies.^39,40^ In sight of the photochemistry of ABs and the recent experimental findings by Schlimm et al.,^27^ triplet excited states and spin–orbit couplings also may be of great interest here, for which our FTDM workflow can be employed as an efficient analysis tool as well. Finally, we emphasize that FTDM-based excited-state characterization is by no means limited to the systems studied in the present work. Apart from AB, we plan to apply our FTDM approach to other functional molecules on noble metals in the near future.

## Supplementary Material



## Data Availability

The data that support the findings of this study are openly available in Zenodo at 10.5281/zenodo.20311224.

## Notes

Computational details for periodic DFT, additional visualized structures, UV/vis absorption spectra and FTDM analyses for different AB isomers, and further excited-state analyses for *trans*-AB dimers on gold and in vacuum (PDF)
