# Supplementary material for: Understanding Molecular Excited States at the Metal–Molecule Interface via Transition Density Matrix Analysis—A Case Study of Azobenzene Thiols on Gold
Source: ACS Phys Chem Au. 2026 Jun 12;6(4):842–57. doi: 10.1021/acsphyschemau.6c00046 (PMC13397451; doi:10.1021/acsphyschemau.6c00046)
Supplement: Supplementary file 1 [file pg6c00046_si_001.pdf]

Supporting Information for:
"Understanding molecular excited states at the metal–molecule interface via transition density matrix analysis — A case study of azobenzene thiols on gold"

Nicolas Jahn[1,*] and Evgenii Titov[1,†]

[1] *University of Potsdam, Institute of Chemistry,*
Karl-Liebknecht-Str. 24-25, 14476 Potsdam-Golm, Germany

(Dated: March 6, 2026)

CONTENTS

S1. Optimization of lattice parameters for bulk gold S2

S2. Additional data for upright *trans*-AB on Au_{25} S3

S3. Additional testing of computational parameters S5

S4. Additional data for other AB isomers S7

S5. Additional data for *trans*-AB dimers S9

* nijahn@uni-potsdam.de

† titov@uni-potsdam.de

S1. OPTIMIZATION OF LATTICE PARAMETERS FOR BULK GOLD

The lattice parameters for bulk gold were optimized employing spin-polarized density functional theory in VASP 5.4.4. The calculation was performed using the projector augmented wave (PAW) method with the TPSS *meta*-GGA functional paired with Grimme's D3 dispersion correction plus Becke–Johnson damping (D3BJ) and standard PBE pseudopotentials (including eleven explicitly treated valence electrons). Standard D3BJ parameters as implemented in VASP 5.4.4 were used for the dispersion correction. Furthermore, a 600 eV plane wave cutoff, a 15x15x15 gamma-centered k-point mesh, first-order Methfessel–Paxton smearing with a smearing parameter of 0.1 eV and a denser DFT grid (PREC=Accurate) have been used. The convergence criteria for energies and forces were set to 10^{-8} eV and 0.001 eV/Å, respectively.

S2. ADDITIONAL DATA FOR UPRIGHT *TRANS*-AB ON AU_{25}

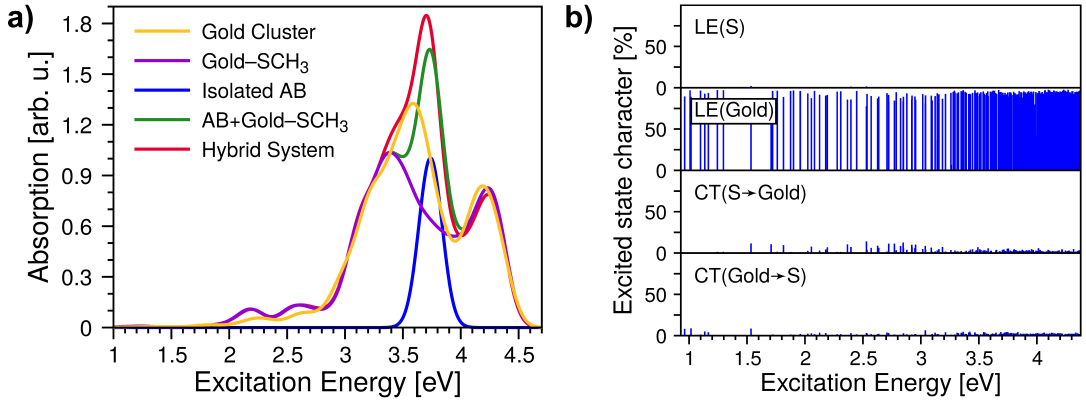

FIG. S1. Additional results for upright *trans*-AB on Au_{25} at the TD-CAM-B3LYP/def2-TZVP level. a) UV/Vis absorption spectrum of the hybrid AB–gold system (red, see Fig. 2a) in comparison to the spectra of the free AB chromophore in vacuum (blue), the Au_{25} cluster with a chemisorbed SCH_3 group (violet) and the bare Au_{25} cluster (yellow). The green line shows the sum of the blue and violet spectra. The bare Au_{25} cluster was treated as a neutrally charged, unrestricted doublet. b) Three fragment FTDM analysis using AB+spacer, binding S atom and Au_{25} fragments. Only local excitations of and charge transfer excitations between the S atom and the gold cluster are shown.

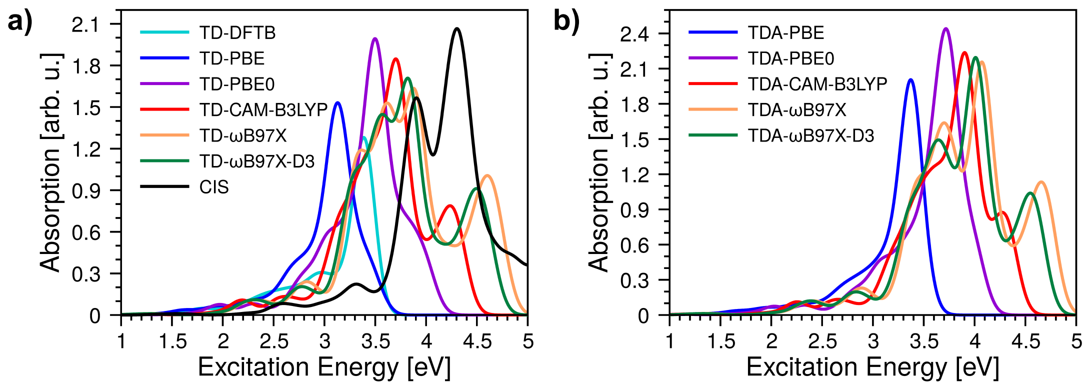

FIG. S2. UV/Vis absorption spectra of upright *trans*-AB with two CH_2 spacer units on Au_{25} employing different computational methods. a) TD-DFT, TD-DFTB and CIS results. b) Results employing the Tamm–Dancoff approximation (TDA). All calculations except for TD-DFTB were performed employing the def2-TZVP basis set. Absorption values for CIS are scaled by a factor of 0.5 to better compare the spectral features of the different methods.

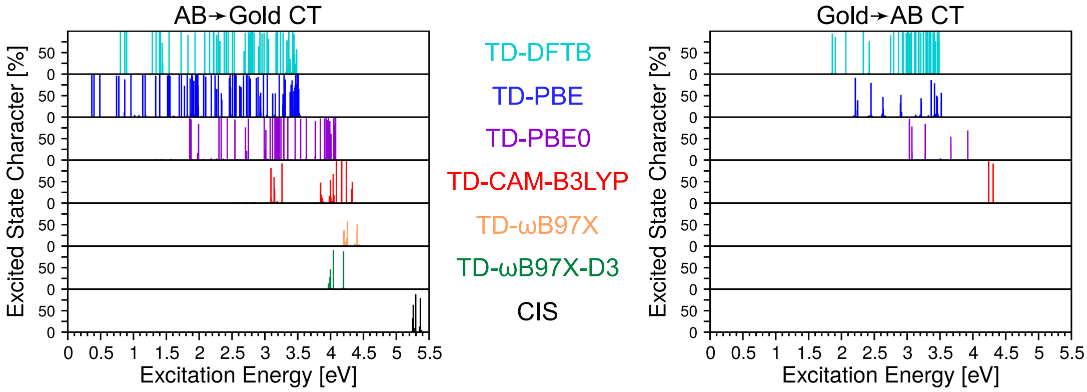

FIG. S3. Charge transfer (CT) excited state analysis of upright *trans*-AB with two CH_2 spacer units on Au_{25} employing different computational methods. Left: AB→gold CT. Right: gold→AB CT. All calculations except for TD-DFTB were performed employing the def2-TZVP basis set. The depicted CT energy ranges per method are limited by the number of calculated excited states as described in the computational details section of the main text.

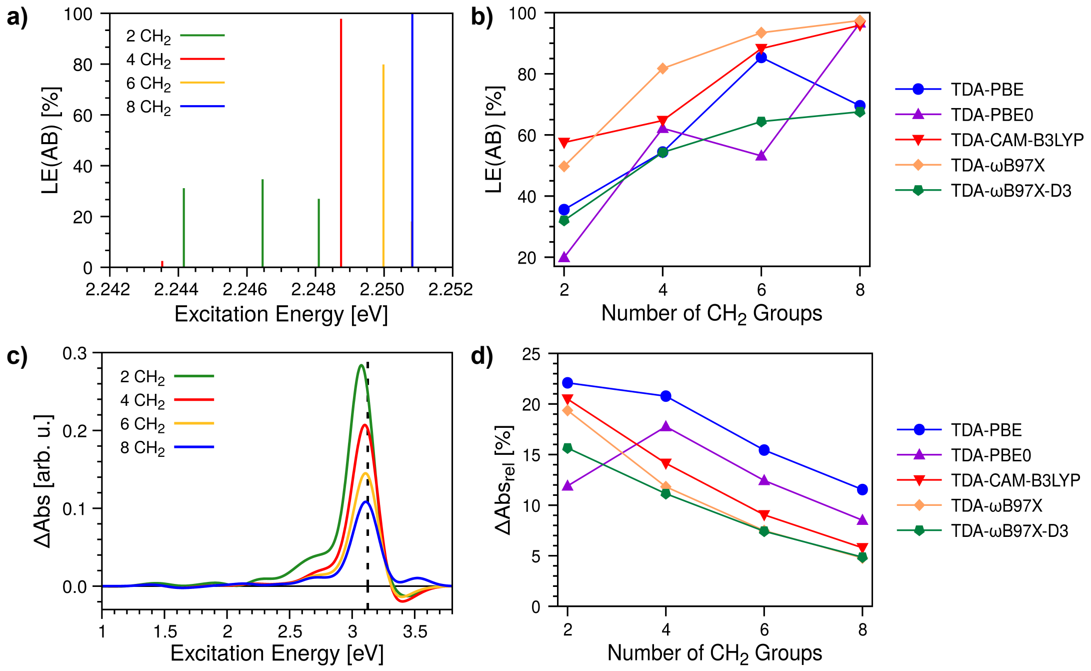

FIG. S4. Additional results for the excited state analysis of upright *trans*-AB on Au_{25} as a function of the alkyl spacer length. a) FTDM analysis of the AB $n\pi^*$ (S_1) state at the TD-PBE/def2-TZVP level of theory. b) Localization of the AB $\pi\pi^*$ state on the molecular part of the hybrid systems as a function of the alkyl spacer length for TDA-DFT. c) Absorption difference spectra at the TD-PBE/def2-TZVP level of theory. The dashed black line shows the excitation energy of the AB $\pi\pi^*$ state. d) Relative absorption increase of the hybrid systems at the AB $\pi\pi^*$ excitation energy for TDA-DFT.

S3. ADDITIONAL TESTING OF COMPUTATIONAL PARAMETERS

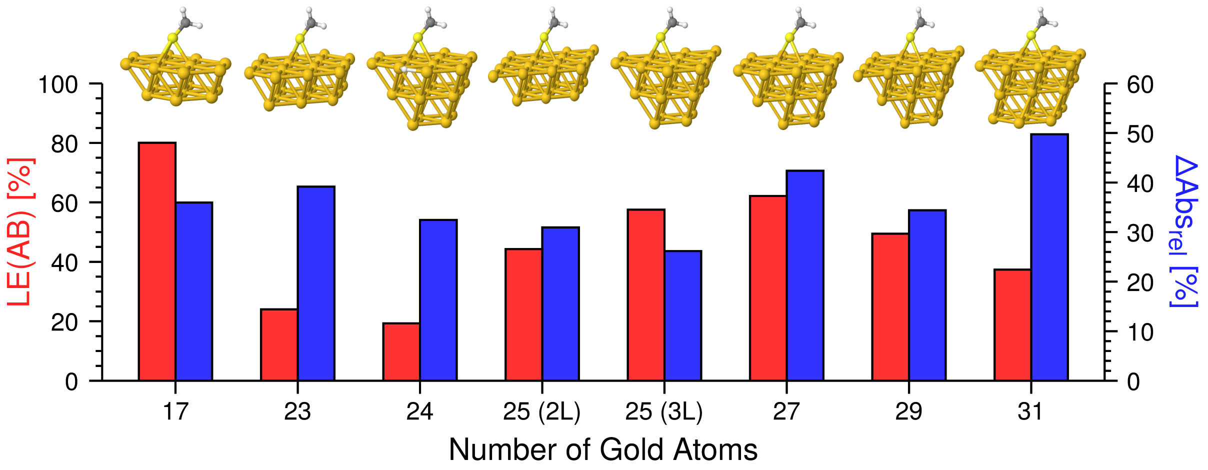

FIG. S5. Influence of the gold cluster form/size on the excited states of upright *trans*-AB with two CH_2 spacer units on gold at the TDA-CAM-B3LYP/def2-TZVP level. LE(AB) values are shown in red and ΔAbs_{rel} in blue. The utilized cluster models are shown above the plot.

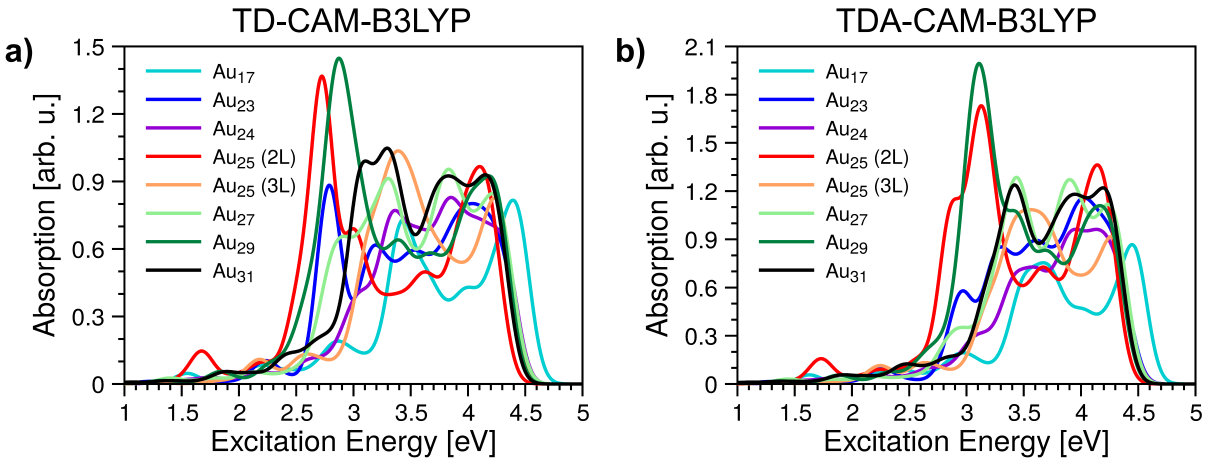

FIG. S6. UV/Vis absorption spectra of different gold clusters with a chemisorbed SCH_3 group. a) TD-CAM-B3LYP/def2-TZVP results. b) TDA-CAM-B3LYP/def2-TZVP results. The corresponding cluster structures are shown in Fig. S5.

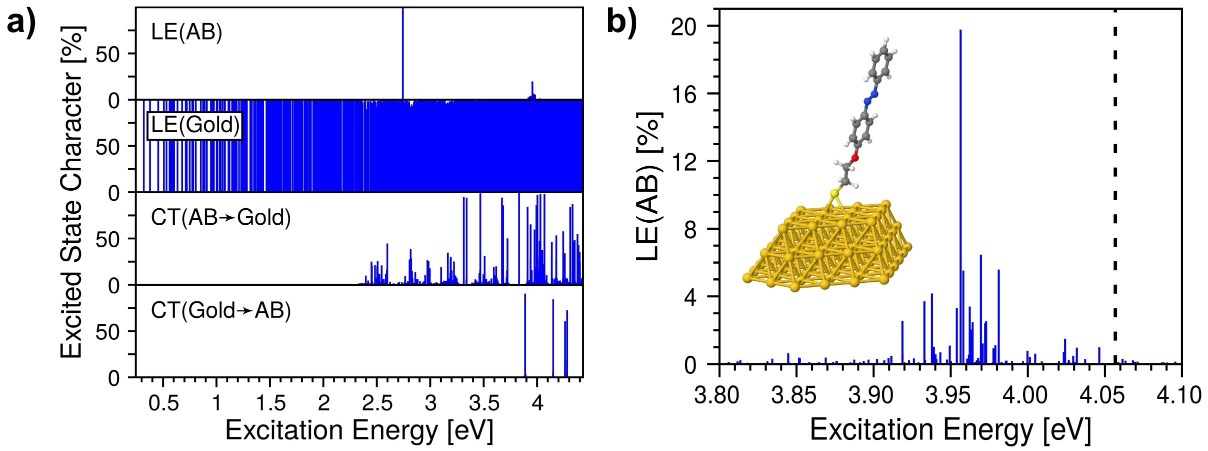

FIG. S7. FTDM excited state analysis of upright *trans*-AB with two CH_2 units on Au_{73} at the TDA-CAM-B3LYP/def2-SVP level. a) Full FTDM plot including all local excitations of and charge transfer contributions between the AB+spacer and gold+S fragments. b) Detailed view of the local AB $\pi\pi^*$ excitations from a). The dashed black line shows the excitation energy of the $\pi\pi^*$ state for the free *trans*-AB molecule at the same level of theory.

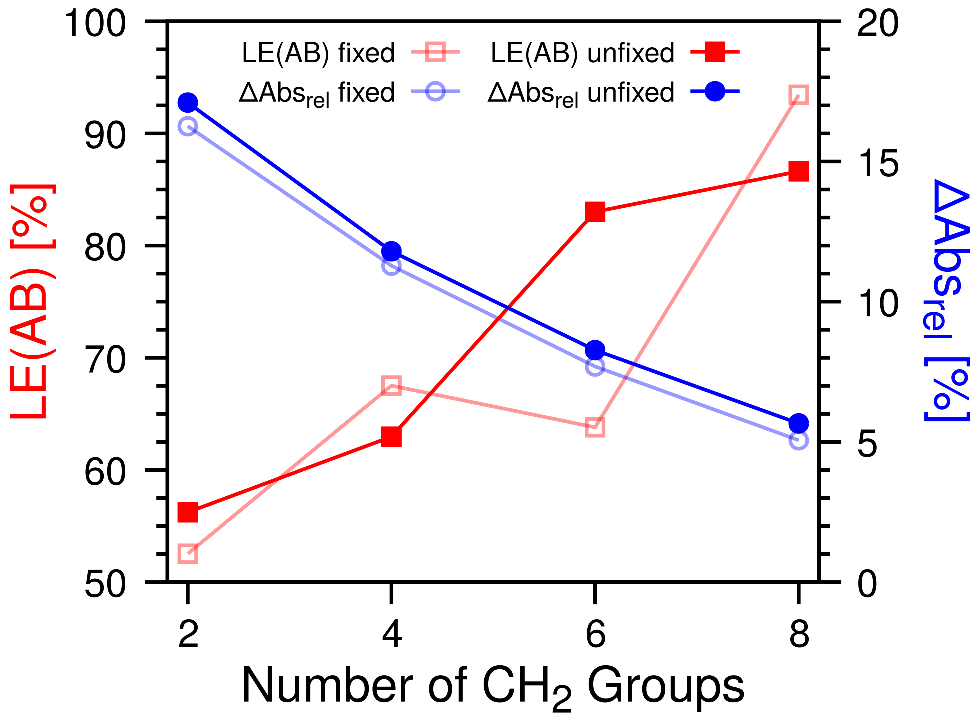

FIG. S8. LE(AB) (red) and ΔAbs_{rel} (blue) values for upright *trans*-AB on Au_{25} as a function of the alkyl spacer length at the TD-CAM-B3LYP/def2-TZVP level. Results for fully frozen gold cluster geometries are labeled as "fixed" and are depicted as semi-transparent, while the first gold layer was included in the original geometry optimizations for the "unfixed" results.

S4. ADDITIONAL DATA FOR OTHER AB ISOMERS

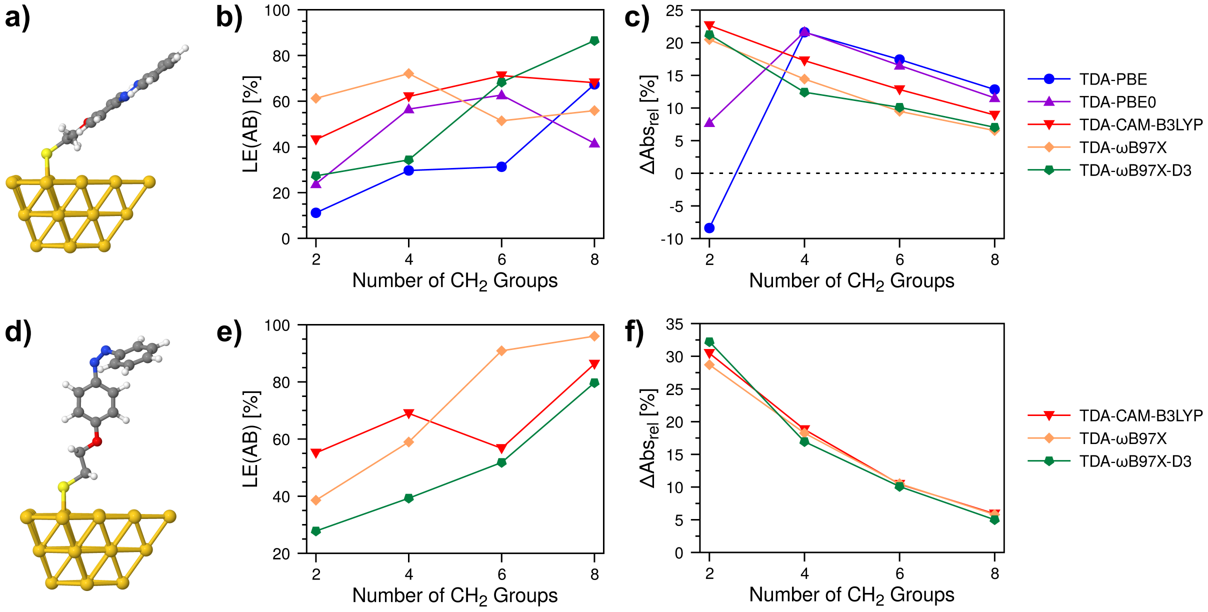

FIG. S9. Excited state analysis of other AB isomers on Au_{25} within the TDA. a-c) Side view, localization of the AB $\pi\pi^*$ excitation and relative absorption increase at the $\pi\pi^*$ excitation energy for flat *trans*-AB. d-f) Results for upright *cis*-AB.

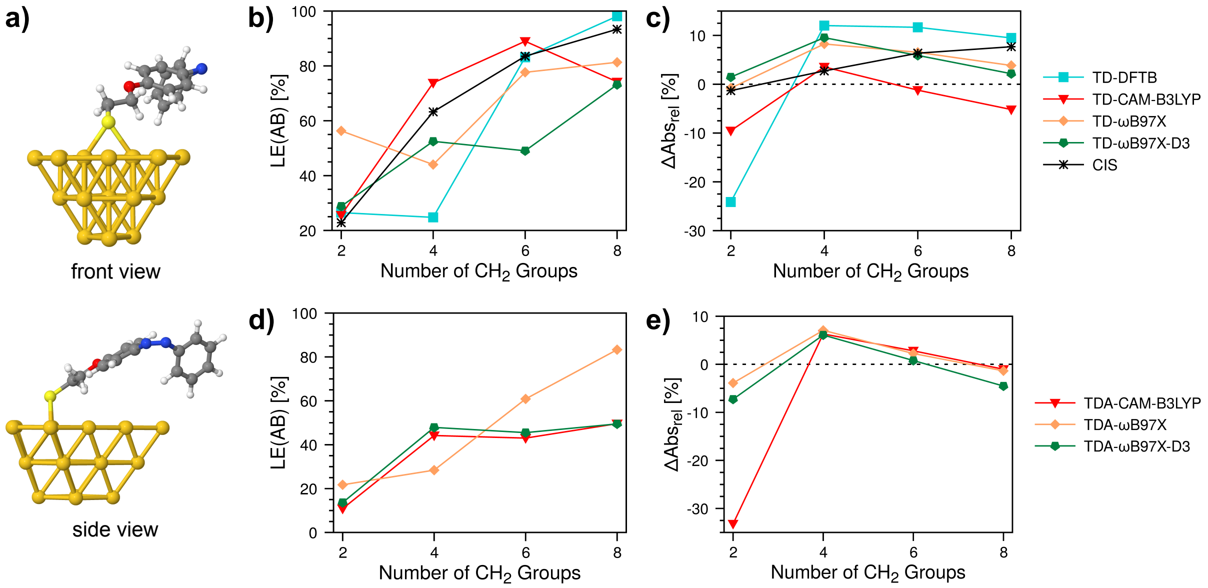

FIG. S10. Excited state analysis of flat *cis*-AB on Au_{25}. a) Front and side view of the AB–gold hybrid system. b), c) Localization of the AB $\pi\pi^*$ excitation and relative absorption increase at the $\pi\pi^*$ excitation energy using TD-DFT, TD-DFTB and CIS. d), e) Results within the TDA.

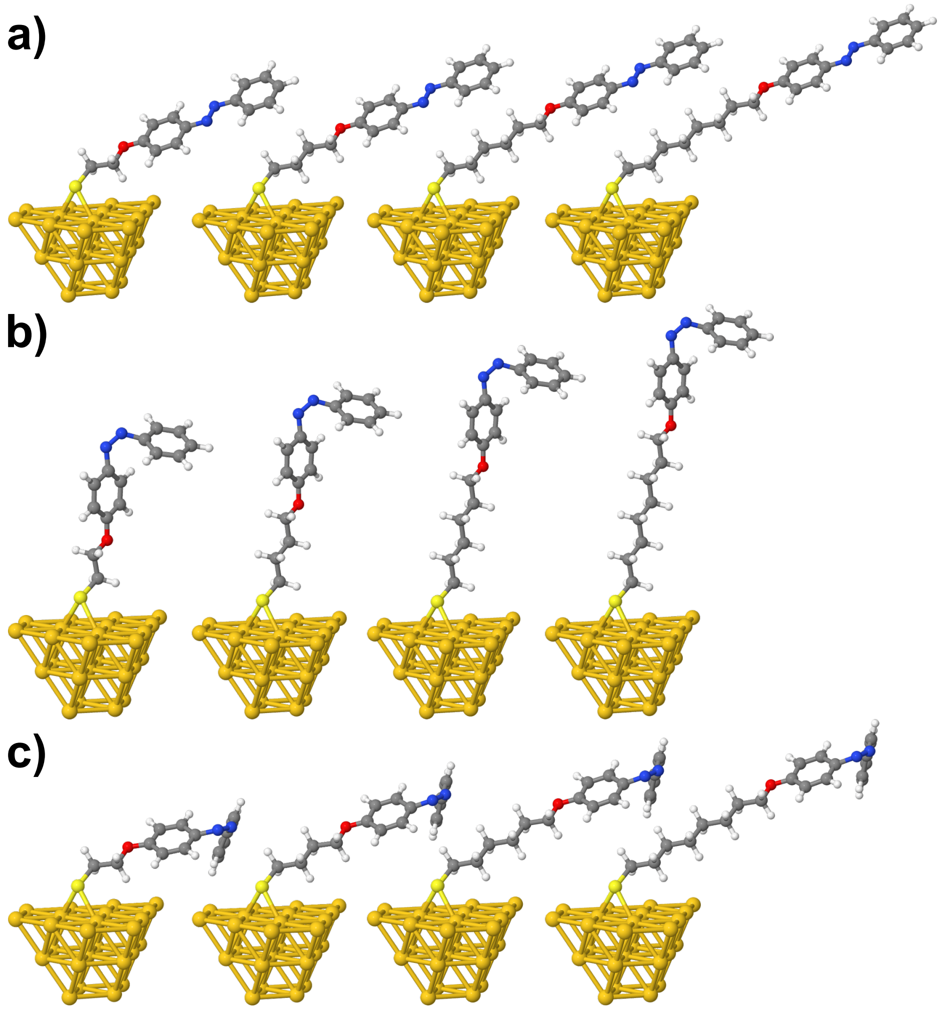

FIG. S11. Overview of the molecular structures for the three additional AB isomers on Au_{25} with four different alkyl spacer lengths. a) flat *trans*-AB. b) upright *cis*-AB. c) flat *cis*-AB.

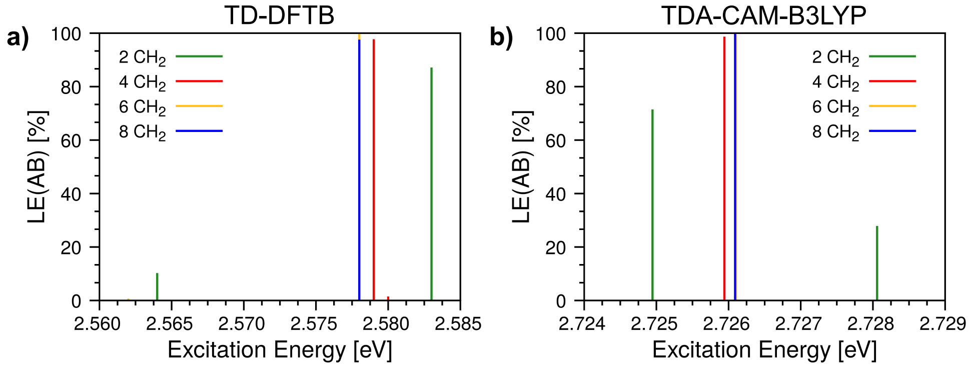

FIG. S12. FTDM analysis of the AB nπ^* (S_1) state for upright *cis*-AB on Au_{25} as a function of the alkyl spacer length. a) Results employing TD-DFTB with the auorg-1-1 parameter set. b) Results at the TDA-CAM-B3LYP/def2-TZVP level. We note that the results for six and eight CH_2 units partially overlap and the former is thus only faintly visible in the graphs.

S5. ADDITIONAL DATA FOR *TRANS*-AB DIMERS

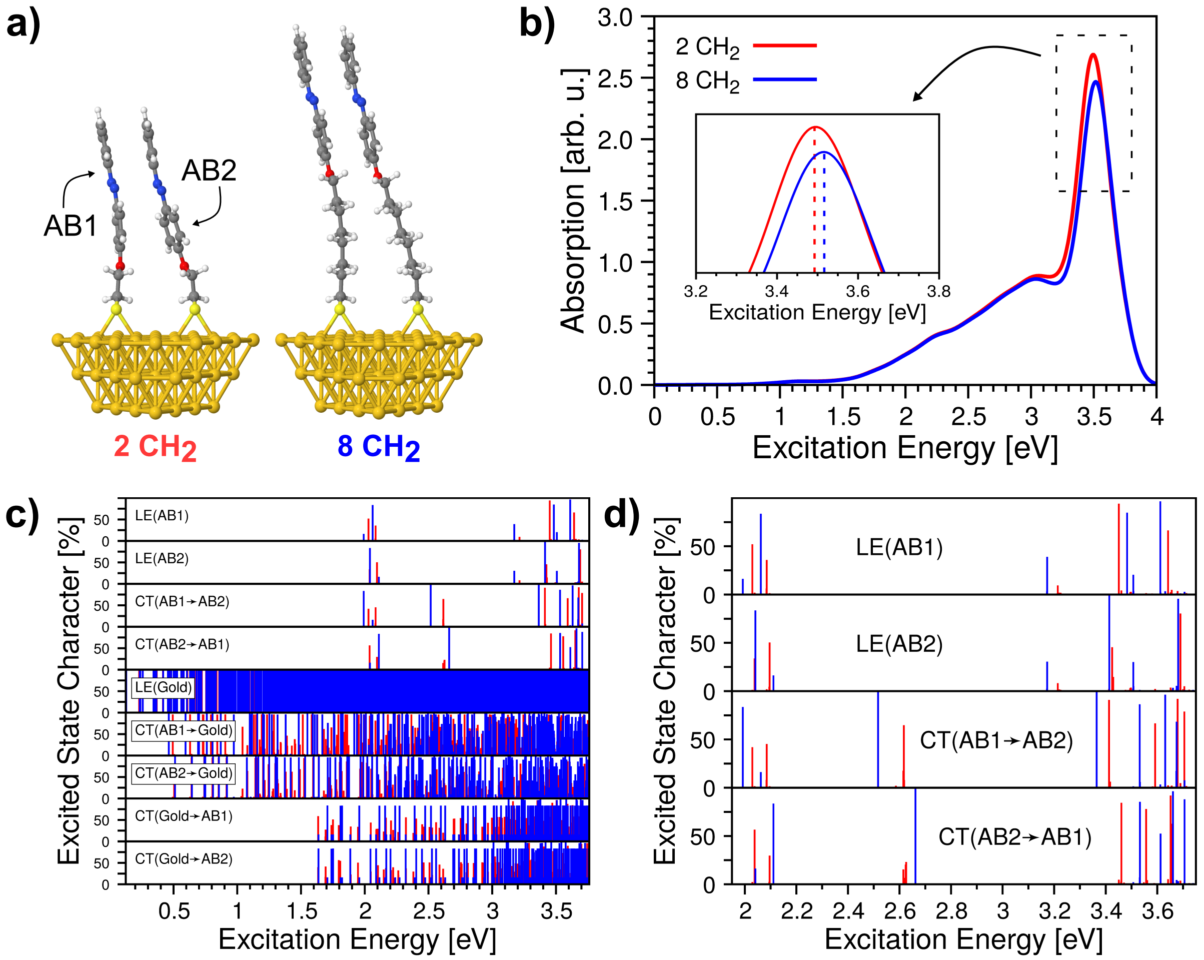

FIG. S13. Excited state analysis of *trans*-AB dimers with two and eight CH_2 units on Au_{64} at the TD-DFTB level. a) TPSS/def2-SVP structures of the two AB-dimer hybrid models. b) UV/Vis absorption spectra of the systems in a). c) Full three fragment FTDM analyses of the structures from a) considering the two *trans*-ABs as individual fragments and the Au_{64} cluster including the binding sulfur atoms as the third fragment. d) Local excitations of and charge transfer contributions between the two ABs from b).

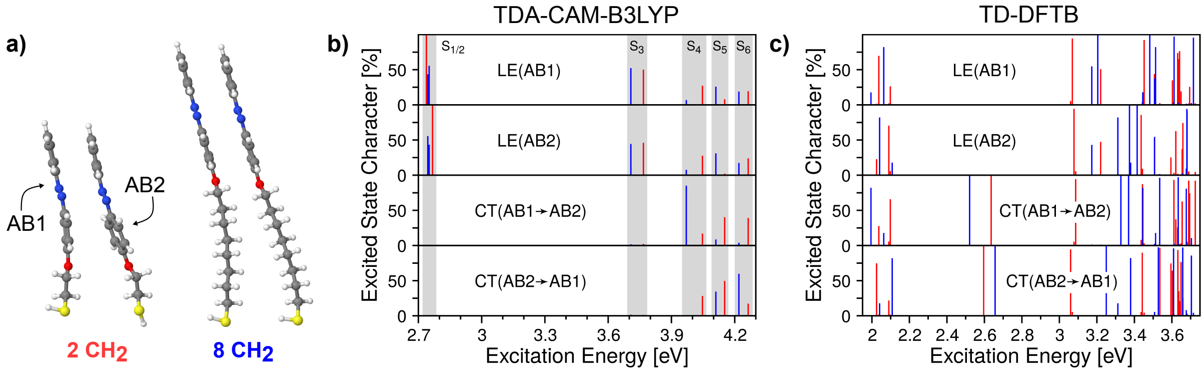

FIG. S14. Excited state analysis of the free *trans*-AB dimer models. a) TPSS-D3BJ/def2-SVP optimized dimer geometries. b) FTDM analysis of the structures in a) at the TDA-CAM-B3LYP/def2-SVP level. c) FTDM analysis employing TD-DFTB. Results for the dimer with two CH_2 units are shown in red and with eight CH_2 units in blue across the Figure.

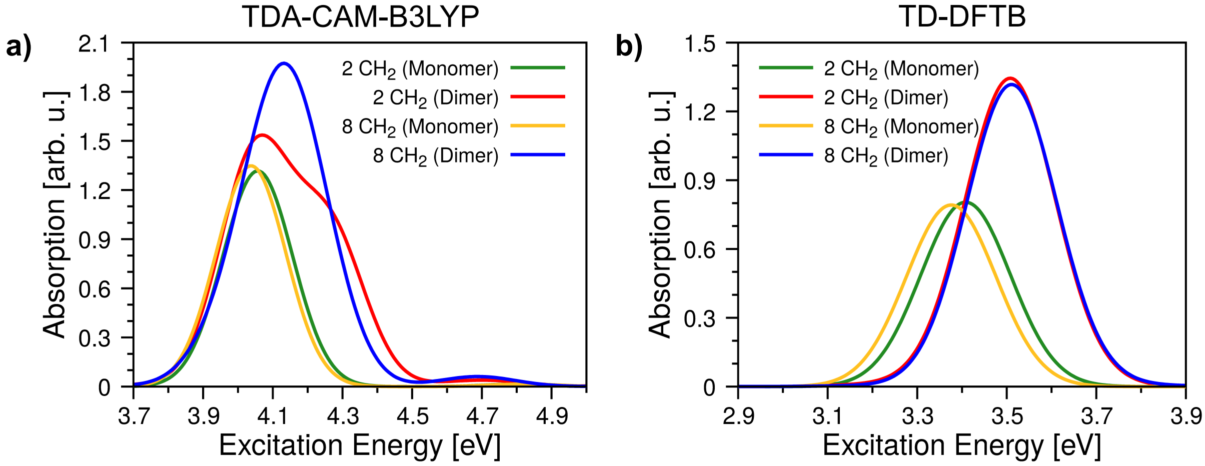

FIG. S15. UV/Vis absorption spectra of the free *trans*-AB dimers in vacuum in comparison to the corresponding *trans*-AB monomers. a) TDA-CAM-B3LYP/def2-SVP results. b) Results at the TD-DFTB level. The TPSS-optimized free dimer geometries are shown in Fig. S14a.
